# Generation of multilineage liver organoids with luminal vasculature and bile ducts from human pluripotent stem cells via modulation of Notch signaling

**DOI:** 10.1186/s13287-023-03235-5

**Published:** 2023-02-03

**Authors:** Hyo Jin Kim, Gyeongmin Kim, Kyun Yoo Chi, Hyemin Kim, Yu Jin Jang, Seongyea Jo, Jihun Lee, Youngseok Lee, Dong-Hun Woo, Choongseong Han, Sang Kyum Kim, Han-Jin Park, Jong-Hoon Kim

**Affiliations:** 1 Laboratory of Stem Cells and Tissue Regeneration, Department of Biotechnology, College of Life Sciences and Biotechnology, Korea University, 145 Anam-Ro, Seongbuk-Gu, Seoul, 02841 South Korea; 2 Department of Predictive Toxicology, Korea Institute of Toxicology, Daejeon, 34114 South Korea; 3 Department of Molecular Biosciences, The University of Texas at Austin, Austin, TX 78712 USA; 4 Department of Stem Cell Biology, NEXEL Co., Ltd, Seoul, 07802 South Korea; 5 College of Pharmacy, Chungnam National University, Daejeon, 34134 South Korea

**Keywords:** Liver organoid, Vasculature, Bile duct, Notch, Hepatic stellate cell, Fibrosis

## Abstract

**Background:**

The generation of liver organoids recapitulating parenchymal and non-parenchymal cell interplay is essential for the precise in vitro modeling of liver diseases. Although different types of multilineage liver organoids (mLOs) have been generated from human pluripotent stem cells (hPSCs), the assembly and concurrent differentiation of multiple cell types in individual mLOs remain a major challenge. Particularly, most studies focused on the vascularization of mLOs in host tissue after transplantation in vivo. However, relatively little information is available on the in vitro formation of luminal vasculature in mLOs themselves.

**Methods:**

The mLOs with luminal blood vessels and bile ducts were generated by assembling hepatic endoderm, hepatic stellate cell-like cells (HscLCs), and endothelial cells derived entirely from hPSCs using 96-well ultra-low attachment plates. We analyzed the effect of HscLC incorporation and Notch signaling modulation on the formation of both bile ducts and vasculature in mLOs using immunofluorescence staining, qRT-PCR, ELISA, and live-perfusion imaging. The potential use of the mLOs in fibrosis modeling was evaluated by histological and gene expression analyses after treatment with pro-fibrotic cytokines.

**Results:**

We found that hPSC-derived HscLCs are crucial for generating functional microvasculature in mLOs. HscLC incorporation and subsequent vascularization substantially reduced apoptotic cell death and promoted the survival and growth of mLOs with microvessels. In particular, precise modulation of Notch signaling during a specific time window in organoid differentiation was critical for generating both bile ducts and vasculature. Live-cell imaging, a series of confocal scans, and electron microscopy demonstrated that blood vessels were well distributed inside mLOs and had perfusable lumens in vitro. In addition, exposure of mLOs to pro-fibrotic cytokines induced early fibrosis-associated events, including upregulation of genes associated with fibrotic induction and endothelial cell activation (i.e., collagen I, *α*-SMA, and ICAM) together with destruction of tissue architecture and organoid shrinkage.

**Conclusion:**

Our results demonstrate that mLOs can reproduce parenchymal and non-parenchymal cell interactions and suggest that their application can advance the precise modeling of liver diseases in vitro.

**Supplementary Information:**

The online version contains supplementary material available at 10.1186/s13287-023-03235-5.

## Background

The liver is a complex organ composed of parenchymal hepatocytes and non-parenchymal cells of distinct embryological origins. Although hepatocytes are responsible for most liver functions [1], non-parenchymal cells such as cholangiocytes, endothelial cells (ECs), hepatic stellate cells (HSCs), and Kupffer cells also play important roles in the normal and diseased liver. Cholangiocytes are biliary epithelial cells that form intrahepatic and extrahepatic bile ducts and were recently identified as a source of pro-inflammatory cytokines and facultative hepatic stem cells in the liver [2–4]. HSCs are specialized liver pericytes that serve as a vitamin A reservoir in the healthy liver [5]. In pathological conditions, activated HSCs are transformed into myofibroblasts that produce a large amount of extracellular matrix (ECM) and pro-inflammatory cytokines that induce wound-healing reactions. However, prolonged activation of HSCs results in liver fibrosis and subsequent cirrhosis [5–7]. Liver sinusoidal ECs are activated in response to pro-inflammatory cytokines and contribute to the progression of liver fibrosis and regeneration by releasing angiocrine growth factors including Wnt2, HGF, TGF*β*, and BMP2 [7]. These non-parenchymal cells also affect entire stages of liver development, including liver bud formation [1, 8–11], by secreting cytokines or transducing direct cell–cell contact-mediated signals such as Notch that modulate vascularization [12–15] and biliary morphogenesis [16, 17].

Technological advances in generating multilineage liver organoids (mLOs) from human pluripotent stem cells (hPSCs) have recently provided an alternative model for investigating liver development and disease [18, 19]. Recent studies demonstrate that mLOs consisting of parenchymal and non-parenchymal cells partially reflect complex interactions between diverse cell types under physiological and pathological conditions in the liver [20, 21]. The first such study generated liver bud-like organoids composed of human induced pluripotent stem cell (hiPSC)-derived hepatic endoderm (HE), primary mesenchymal stem cells, and human umbilical vein endothelial cells [22]. Based on this approach, subsequent studies generated different types of mLOs consisting of various combinations of cell types obtained directly from primary liver tissue [23–27] or entirely from hPSCs for modeling liver diseases [20, 28]. Recent studies produced mLOs containing HSCs, ECs, or Kupffer cells to model liver diseases, including non-alcoholic steatohepatitis [29–31]. However, most studies focused on the vascularization of mLOs in host tissue after transplantation in vivo, with relatively little information available on the in vitro formation of luminal vasculature in mLOs themselves. Other studies generated organoids with bile duct structures and biliary cysts from human hepatic progenitors [32] or hPSCs [33], but these organoids did not contain HSCs and ECs. Overall, despite recent advances in hPSC and organoid technologies, incorporating multiple non-parenchymal cell types and inducing structural maturation of mLOs with both luminal blood vessels and bile ducts remains a major challenge.

We previously showed that hepatic functions of hPSC-derived cells can be increased by generating 3D hepatic spheroids [34, 35]. We also demonstrated that genome-edited mLOs derived from a patient with hemophilia A (HA) secrete significant amounts of blood coagulation factor, FVIII [36]. In the present study, we focused on the generation of both luminal vasculature and bile ducts in mLOs entirely from hPSCs in vitro. We found that the inclusion of HscLCs and regulation of Notch signaling during mLO assembly and maturation inhibited apoptotic cell death and promoted both vascularization and biliary duct formation in mLOs. Furthermore, the mLOs produced capillary-like vessels with perfusable lumens and were sensitive to fibrosis-associated cytokines, reflecting multiple early aspects of fibrotic induction.

## Materials and methods

### Generation and culture of hPSC-derived mLOs

The generation of mLOs is summarized in Fig. 2A. BG01-derived HEs, ECs, and HscLCs were dissociated into single-cell suspensions by treatment with TrypLE (12604-021, Gibco) for 3 min and collected using DMEM (12100-046, Gibco) containing 10% FBS (16000-044, Gibco). 1.5 × 10^4^ HE, 3 × 10^4^ ECs, and 5 × 10^3^ HscLCs were seeded together in ultra-low attachment 96-well plates (ULA PrimeSurface® 96U, MS-9096UZ, SIMADZU) in cold multilineage liver organoid differentiation medium [37–40] [DM, advanced DMEM/F12 media supplemented with 1 × HEPES (15630-080, Gibco), 1 × GlutaMAX (35050-061, Gibco), 1 × penicillin/streptomycin (15140-122, Gibco), 0.1 mg/ml BSA (A7096, Sigma), 1 × B27 supplement minus vitamin A (12587-010, Gibco), 25 ng/ml BMP7 (120-03p, Peprotech), 100 ng/ml FGF19 (100-32, Peprotech), 25 ng/ml HGF (CYT-244, Prospec), 10 nM (Leu15)-Gastrin I (G9145, Sigma), 1.25 mM N-acetyl-L-cysteine (A9165, Sigma), 0.5 μM A83-01 (2939, Tocris Bioscience), 10 μM DAPT (ab120633, Abcam), 3 μM dexamethasone (D4902, Sigma), 0.566 mg/ml growth factor-reduced Matrigel (M354230, Corning), 100 ng/ml VEGF-165 (100-20, Peprotech), 50 ng/ml bFGF (100-18b, Peprotech), and 10 μM ROCK inhibitor Y27632 (1254, Tocris Bioscience)]. On days 1 and 3, cold DM was added to the organoid culture. Five days after aggregation, mLOs were transferred to ultra-low attachment 24-well plates (3473, Corning) and further cultured in DM. The medium was replaced every 3 days. To optimize the ratio and the composition of cell types in mLOs (total of 5 × 10^4^ cells), different ratios (HE/EC/HscLC, 3:6:1; 5:4:1; 7:2.5:0.5) and combinations (HE + EC + HscLC; HE + HscLC; HE only) of cell types were assembled in ULA 96-well plates and cultured as described above.

### Gene expression analysis

Total RNA was isolated from 2D cells and organoids using TRIzol (15596-018, Invitrogen) and homogenizers. cDNA was synthesized using a reverse-transcription system (Thermo Fisher Scientific). Quantitative reverse-transcription polymerase chain reaction (qRT-PCR) was performed using a CFX-96 real-time PCR detection system (Bio-Rad) in triplicate with iQ™ SYBR Green SuperMix (170-8882, Bio-Rad). Primer sequences are presented in [Additional file 1: Table S1]. Relative expression levels of target genes were normalized to the level of glyceraldehyde 3-phosphate dehydrogenase (*GAPDH*) or beta-actin (*ACTB*) mRNA.

### Immunofluorescence staining of 2D cells and organoid cryosections

2D cells were fixed in 4% paraformaldehyde solution for 20 min at room temperature and then washed three times with PBS. Organoids were fixed in 4% paraformaldehyde solution for 1 h at room temperature and incubated overnight at 4 °C in 20% sucrose for cryo-protection. Fixed organoids were embedded in optimum cutting temperature (OCT) compound (4283, Tissue-Tek) and cut into 30- or 50-μm frozen sections. Fixed cells or organoid cryosections were incubated with blocking solution [10% donkey serum (ab7475, Abcam) in PBS with or without 0.3% Triton X-100] for 45 min at room temperature and then incubated with primary antibodies (Additional file 1: Table S2) overnight at 4 °C. After washing several times with PBS, cells were incubated with Alexa Flour 488-, 568-, 594-, or 647-conjugated secondary antibodies (Additional file 1: Table S2) for 1.5 h at room temperature in the dark. Cells were rinsed several times with PBS, and nuclei were stained with 1 μg/ml 4’,5-diamidino-2-phenylindole (DAPI, D8417, Sigma) for 10 min at room temperature. Fluorescence images were obtained using a confocal laser scanning microscope (LSM800, Carl Zeiss). Image quantification was performed using ImageJ.

### Whole-mount immunofluorescence staining and clearing of organoids

Organoids were washed three times with cold PBS and fixed in 4% paraformaldehyde solution for 1 h at room temperature on a rocker. Fixed organoids were rinsed with PBS three times and incubated with organoid-blocking solution [10% donkey serum (ab7475, Abcam), 1% BSA (BSA 0.1, Bovogen), and 0.3% Triton X-100 in PBS] for 3 h at room temperature. The organoids were probed with primary antibodies (Additional file 1: Table S2) overnight at 4 °C on a rocker. After washing three times with PBS, organoids were incubated with Alexa Flour 488-, 568-, or 647-conjugated secondary antibodies (Additional file 1: Table S2) for 3 h at room temperature. Organoids were washed three times with PBS, and nuclei were stained with 1 μg/ml DAPI (D8417, Sigma) in PBS for 1 h. After washing several times with PBS, organoids were dehydrated by serial incubation with 30% and 50% ethanol in PBS and 70%, 90%, and 100% ethanol in distilled water for 20 min each at room temperature. Dehydrated organoids were then cleared with CytoVista 3D Cell Culture Clearing Reagent (V11326, Invitrogen) for 3–4 days in the dark. Fluorescence images were obtained using a confocal laser scanning microscope (LSM800, Carl Zeiss). Image quantification was performed using ImageJ and AngioTool.

### Live perfusion of organoids with UEA-I lectin

Organoids were washed twice with cold organoid basal media (advanced DMEM/F12 media containing 0.1 mg/ml BSA, 1 × HEPES, 1 × penicillin/streptomycin, and 1 × GlutaMAX) and transferred to a μ-Slide Angiogenesis Glass Bottom (81507, iBiDi) for live time-lapse imaging. Organoids were treated with 20 μg/ml rhodamine-conjugated Ulex Europaeus Agglutinin l (UEA-I-rhodamine, RL-1062–2, Vector Laboratory) in organoid basal media. Live time-lapse images were obtained by a confocal laser scanning microscope (LSM800, Carl Zeiss). For whole-mount immunofluorescence staining after live perfusion, organoids were incubated with 20 μg/ml UEA-I-rhodamine in organoid basal media at 37 °C for 30 min. After washing three times with cold PBS, organoids were fixed using 4% paraformaldehyde in PBS for 1 h, and whole-mount staining and clearing were performed as described above.

### Statistical analysis

Statistical analyses of qRT-PCR data and quantification data from immunofluorescence images were performed using GraphPad Prism software. All data are presented as mean ± standard deviation (SD) from three independent experiments performed in triplicate unless otherwise indicated. Statistical significance was assessed using unpaired Student’s *t* tests. *p* < 0.05 was considered statistically significant.

Details of additional experimental methods and data are available in the supporting information.

## Results

### Generation and characterization of multiple cell types from hPSCs for the generation of mLOs

**Fig. 1:**
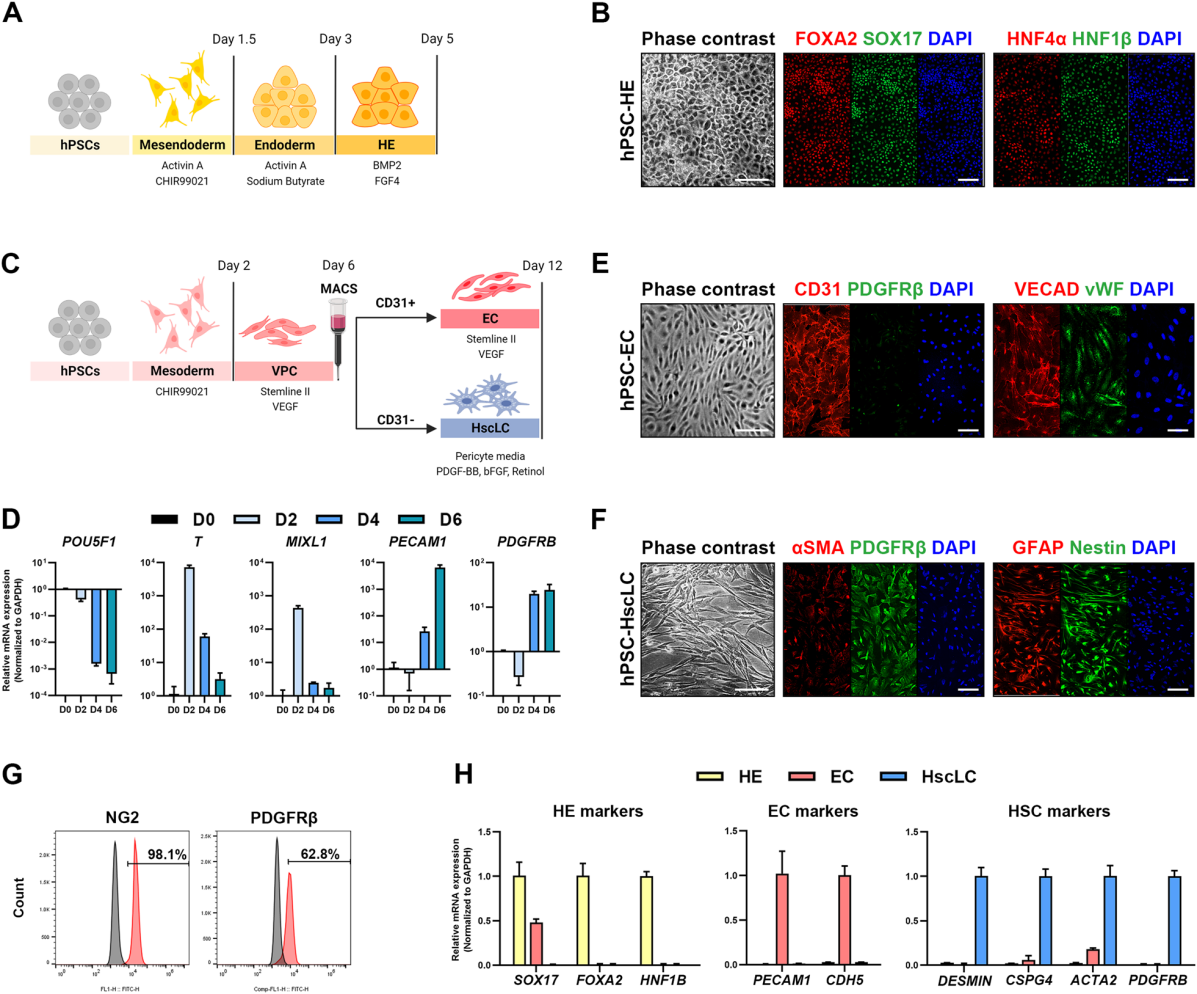
Generation and characterization of HE, ECs, and HscLCs from hPSCs. **A** Schematic protocol for differentiation of HE from hPSCs (created with BioRender.com). **B** Phase-contrast and immunofluorescence images of endodermal markers (FOXA2 and SOX17) and HE markers (HNF4*α* and HNF1*β*) in hPSC-derived HE. Scale bar, 100 μm. **C** Schematic protocol for differentiation of EC and HscLCs from hPSCs (created with BioRender.com). **D** qRT-PCR analysis of genes associated with pluripotency (*POU5F1*), mesendoderm (*T* and *MIXL1*), endothelium (*PECAM1*), and pericytes (*PDGFRB*) in differentiating vascular progenitor cells on the indicated days of differentiation. Data are expressed as mean ± SD (*n* = 3, normalized to *GAPDH*). **E**, **F** Phase-contrast and immunofluorescence images of EC markers (CD31, VECAD, and vWF) and HSC markers (*α*-SMA, PDGFR*β*, GFAP, and Nestin) in hPSC-derived ECs and HscLCs. Scale bar, 100 μm. **G** Flow cytometry analysis of HSC markers NG2 and PDGFR*β* in hPSC-derived HscLCs. **H**, qRT-PCR analysis of genes associated with HE, ECs, and HSCs in hPSC-HE, ECs, and HscLCs at the end of differentiation. Data are expressed as mean ± SD (*n* = 3, normalized to *GAPDH*)

To generate liver organoids consisting of different cell types, we first differentiated hepatic endoderm (HE), ECs, and HscLCs from hPSCs. Schematic depictions of the generation of HE, ECs, and HscLCs from hPSCs are shown in Fig. 1A, C. HE and ECs were generated using our previously described protocols [34-36, 41, 42]. hPSC-derived HE contained a homogeneous population of epithelial cells expressing endoderm makers [forkhead box A2 (FOXA2) and SRY-box 17 (SOX17)] and early hepatic markers [hepatocyte nuclear factor 4-alpha (HNF4*α*) and hepatocyte nuclear factor 1-beta (HNF1*β*)] (Fig. 1B). Flow cytometry analysis revealed that 95% and 96.9% of all cells expressed alpha-fetoprotein (AFP) and epithelial cell adhesion molecule (EPCAM), which are markers of early hepatic progenitors (Additional file 1: Fig. S1A).

EC and pericytes are derived from a common set of mesodermal progenitor cells [43–45]. During EC differentiation, Magnetic-activated cell sorting (MACS)-purified CD31 + cells were further differentiated into ECs in Stemline II media supplemented with VEGF-165 for 6 days (Fig. 1C). The remaining CD31- fraction was used to produce HscLCs in the culture medium specially formulated to grow pericytes in the presence of PDGF-BB and bFGF [43, 44]. As hPSCs progressively committed to mesoderm and vascular progenitor cells, the pluripotency marker *POU5F1* (*OCT4*) was downregulated concomitantly with transient upregulation of mesendodermal markers *T* and *MIXL1* on day 2 of differentiation (Fig. 1D). Expression of the endothelial marker *PECAM1* and pericyte marker *PDGFRB* increased upon differentiation into ECs and HscLCs (Fig. 1D). At the end of differentiation (day 12), hPSC-derived ECs expressed different EC markers including CD31, vascular endothelial cadherin (VECAD), and von Willebrand factor (vWF) (Fig. 1E). Most (~ 99.9%) hPSC-derived ECs expressed CD31 as determined by flow cytometry analysis (Additional file 1: Fig. S1B) and were negative for a mural cell marker, platelet-derived growth factor receptor-beta (PDGFR*β*) (Fig. 1E). HSCs are specialized pericytes residing in the liver whose differentiation and maturation require retinoic acid signaling [5, 46–48]. To endow the MACS-sorted CD31- cell fraction with pericyte phenotypes, CD31- cells were differentiated in the pericyte medium supplemented with 10 ng/ml PDGF-BB and 10 ng/ml bFGF in the presence of retinol for an additional 6 days. After a total of 12 days of differentiation, MACS-sorted CD31- cells expressed multiple pericyte or HSC markers including *α*-smooth muscle actin (*α*-SMA), and PDGFR*β*, and also expressed other genes, glial fibrillary acidic protein (GFAP) and Nestin, which are expressed in HSCs [46, 49, 50] (Fig. 1F). FACS analysis indicated that CD31- cells predominantly differentiated into HscLCs expressing neural/glial antigen 2 (NG2) (98.1%) and PDGFR*β* (62.8%) (Fig. 1G). CD31- cell-derived HscLCs exhibited upregulation of genes associated with HSC activation, *ACTA2* (*α*-SMA) and *NESTIN*, as previously reported [51, 52]*,* along with morphological changes upon 2 days of treatment with the pro-inflammatory cytokine TGF*β*1 (Additional file 1: Fig. S2A, B). hPSC-derived HE, ECs, and HscLCs preferentially expressed each lineage marker at the mRNA level (Fig. 1H).

### HscLCs support the generation and growth of mLOs

mLOs were recently demonstrated to produce blood vessels that join to host vasculature following transplantation [22, 23, 28, 53]. However, most liver organoids lack functional vasculatures in vitro and harbor scattered ECs or EC networks, hampering accurate modeling of tissue architecture and diseases. Pericytes are vascular mural cells that promote the formation and maturation of functional vasculature [54–56]. Liver-specific pericytes, HSCs, are closely associated with liver sinusoidal ECs of the liver lobule and contribute to liver development and tissue regeneration after injury [5–7]. Thus, we hypothesized that hPSC-derived HscLCs promote functional maturation of mLOs harboring vascular networks.

**Fig. 2:**
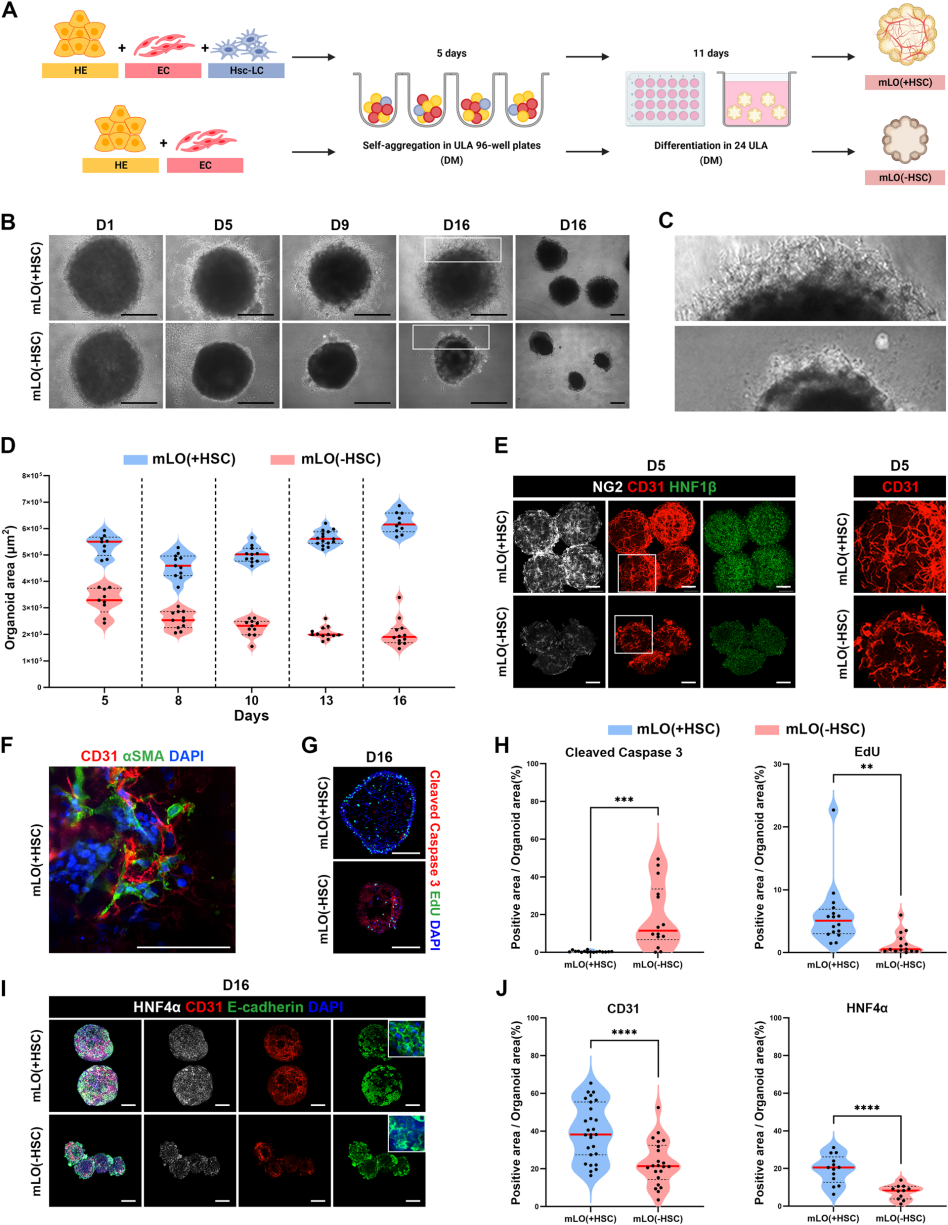
Characterization of mLOs consisting of hPSC-derived HE, ECs, and HscLCs. **A** Schematic protocol for self-aggregation and differentiation of mLOs. mLOs were generated and differentiated with or without HscLCs for 16 days in DM (created with BioRender.com). **B** Phase-contrast images of mLOs generated with [mLO(+ HSC)] or without [mLO(-HSC)] HscLCs on the indicated days of differentiation. Enlarged images showing the periphery of organoids at day 16 are shown separately in **C** Scale bar, 200 μm. **D** Violin plot showing the size distributions of mLO(+ HSC) and mLO(-HSC) on the indicated days of differentiation. Each black dot represents a single organoid. **E** Whole-mount immunofluorescence images of NG2 (pericytes), CD31 (ECs), and HNF1*β* (HE) in mLOs at the end of self-aggregation (day 5). High-magnification images of the boxed areas (CD31) are shown separately in the right panels. Scale bar, 200 μm. **F** Representative immunostaining image showing close association between CD31 + ECs and *α*SMA + HscLCs in mLO(+ HSC) on day 16. Scale bar, 50 μm. **G** Immunofluorescence images of cleaved caspase-3 and EdU in cryo-sectioned mLOs after 16 days of differentiation. mLOs were pre-incubated with EdU for 6 h before fixation. Scale bar, 200 μm. Quantification of immunoreactive signals is shown in **H**. Each black dot represents the percent positive area of the immunoreactive signal in each mLO. **p < 0.01 and ***p < 0.001 by unpaired *t* tests. **I** Whole-mount immunofluorescence images of HNF4*α* (HLCs), CD31, and E-cadherin (hepatic epithelium) in mLOs after differentiation (day 16). The insets show high-magnification images of E-cadherin staining. Scale bar, 200 μm. **J** Quantification of immunoreactive signals in (**I**). Each black dot represents the percent positive area of the immunoreactive signal in each mLO. ****p < 0.0001 by unpaired *t* test

To address this issue, we differentiated HE, ECs, and HscLCs from hPSCs and assembled them in ULA 96-well round-bottomed plates using three different ratios (Fig. 2A and Additional file 1: Fig S3). The assembly ratio of three cell types was optimized (HE/EC/HscLC, 3:6:1) to promote in vitro vascularization of mLOs, while maintaining hepatic epithelium in the differentiation medium (DM) (Additional file 1: Fig. S3). To support the survival and maturation of ECs during the following period of mLO generation (16 days), we also optimized the DM previously used to produce liver organoids from liver tissue and iPSCs [37–39] by adding VEGF and bFGF [54, 55]. mLOs were produced with or without HscLCs in ULA 96-well plates in DM for 5 days and further differentiated in ULA 24-well plates for 11 days using the same culture medium. hPSC-derived cells spontaneously adhered to each other and formed compact spherical clusters without forced aggregation in both mLOs with (+ HSC) and without (-HSC) HscLCs within 1 day in ULA 96-well plates (Fig. 2B). Interestingly, over the 16 days of culture, mLOs containing HscLCs [mLO(+ HSC)] showed presumptive sprouting of EC networks at the periphery of organoids, which was not observed in mLOs devoid of HscLCs [mLO(-HSC)] (Fig. 2B, C). In addition, a decrease in organoid size was found for mLO(-HSC), whereas organoid size increased gradually after day 8 of culture for mLO(+ HSC) (Fig. 2D).

After 5 days of self-aggregation and differentiation, NG2 + HscLCs were predominantly observed in mLO(+ HSC), but only weak NG2 signals were detected in mLO(-HSC) (Fig. 2E, white). We also found multiple *α*-SMA + HscLCs that were preferentially positioned close to CD31 + ECs in mLO(+ HSC) (Fig. 2F). Importantly, CD31 + vascular networks were much longer and thicker in mLO(+ HSC) compared with mLO(-HSC) (Fig. 2E, red; enlarged images of the boxed area are shown separately in the right panel). After a total of 16 days of differentiation, active caspase-3 and 5-ehynyl-2’-deoxyuridine (EdU) labeling showed that apoptotic events were markedly reduced, and proliferation was increased in mLO(+ HSC) compared with mLO(-HSC) (Fig. 2G, H). The numbers of cells expressing CD31, epithelial cadherin (E-cadherin), and HNF4*α* were significantly higher in mLO(+ HSC) than in mLO(-HSC) (Fig. 2I, J), even though the same number of ECs and HE was used to initiate the generation of mLOs in both groups. These results suggest that the incorporation of HscLCs is essential for the survival and growth of mLOs, particularly for in vitro culture requiring long-term evaluation.

### Regulation of Notch signaling for bile duct and vascular formation in mLOs

**Fig. 3:**
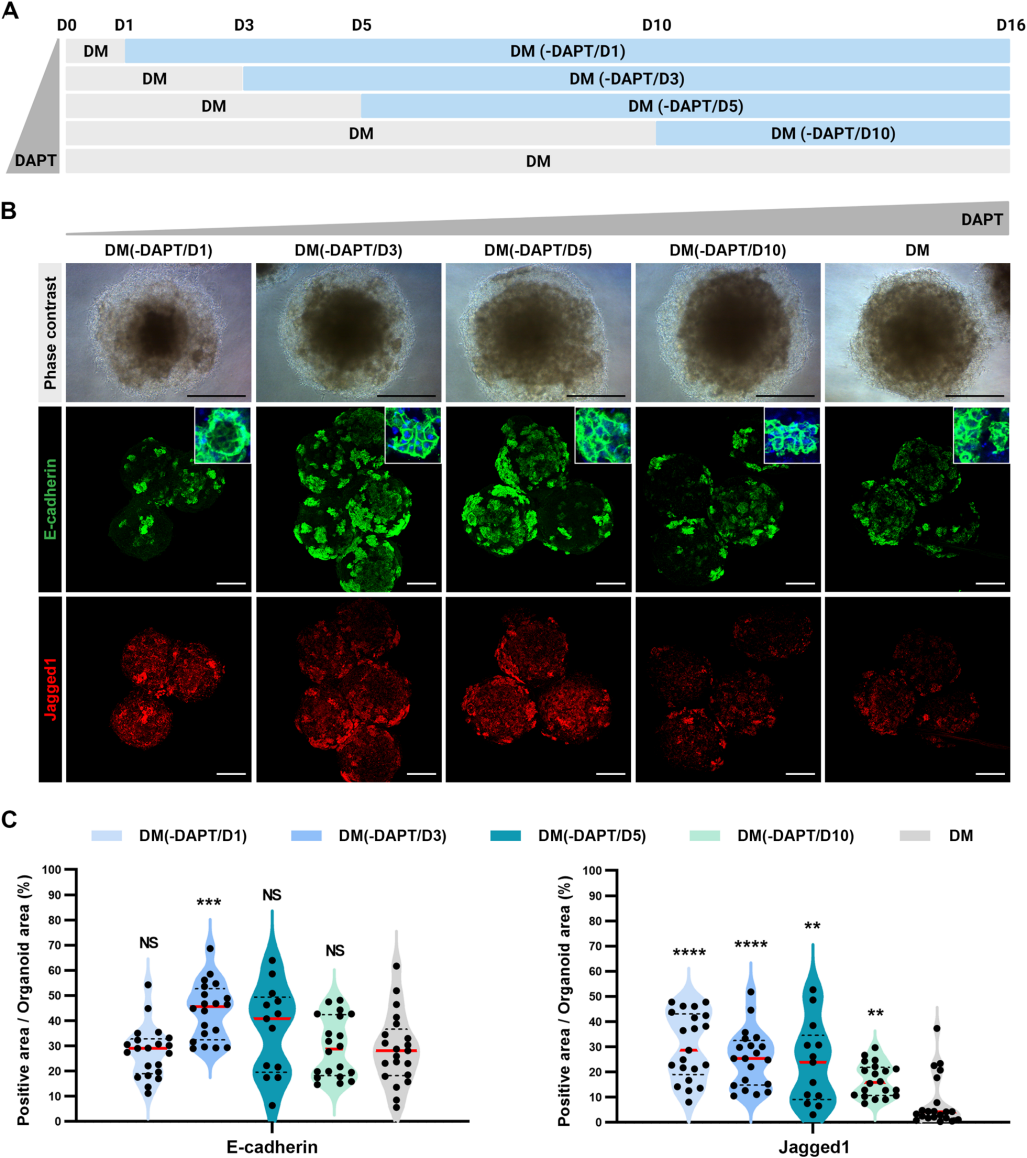
Optimization of Notch signaling activation for generating mLOs. **A** Schematic timeline of the differentiation of mLOs for 16 days in DAPT-containing DM or DM lacking DAPT [DM(-DAPT)]. Days of DAPT removal are indicated in parentheses from day 1 (-DAPT/D1) to 10 (-DAPT/D10) of differentiation. **B** Phase-contrast and immunofluorescent images of E-cadherin (hepatic epithelium) and Jagged 1 (Notch ligand) in mLOs after treatment with DAPT at different time points of differentiation. The insets show high-magnification images of E-cadherin staining. Scale bar, 200 μm. **C** Quantification of immunoreactive signals for E-cadherin and Jagged 1 in B. Each black dot represents the percent positive area of the immunoreactive signal in each mLO. **p < 0.01, ***p < 0.001, and ****p < 0.0001 compared to DM by unpaired *t* tests

Although HscLCs supported the growth of mLOs with vascular structures, bile ducts were not observed in mLOs, suggesting that further optimization of culture conditions is required to produce mLOs representing liver tissue. Notch signaling regulates angiogenic sprouting via lateral inhibition of endothelial stalk cells [12–14] and promotes bile duct morphogenesis [16, 17]. To enrich mature hepatocytes in liver organoids, previous studies used DM containing a Notch signaling inhibitor, DAPT, that promotes preferential differentiation of bipotent hepatoblasts into hepatocytes rather than bile duct cells [37–39]. Notch inhibitors also directly inhibit bile duct differentiation in 2D [57] or 3D [58, 59] culture of hepatic progenitors. Therefore, we hypothesized that the absence of duct-like structures in mLO(+ HSC) is due to the presence of DAPT in DM. We thus investigated the optimum time window of DAPT withdrawal for inducing both bile ducts and vasculature during the generation of mLOs (Fig. 3A). The Notch ligand Jagged1 is essential for the development of intrahepatic bile ducts, and inhibition of Jagged1-mediated Notch signaling or mutation of *JAG1* disrupts biliary development and regeneration and leads to Alagille syndrome [17, 60, 61]. As the duration of DAPT treatment was extended, the Jagged1 + area in mLOs decreased (Fig. 3B, C). E-cadherin is expressed in hepatocytes and biliary ductal cells [62]. The percentage of E-cadherin + epithelial cells was highest in mLOs generated in the differentiation medium devoid of DAPT from day 3 [DM(-DAPT/D3)] or day 5 [DM(-DAPT/D5)] of differentiation (Fig. 3B, C). qRT-PCR analysis showed that the expression of hepatocyte marker genes *CEBPA* and *ALB* tended to increase, whereas the expression of biliary genes *ONECUT1* and *JAG1* tended to decrease as the duration of DAPT treatment increased (Additional file 1: Fig. S4A). Statistical analyses showed that the expression *JAG1* was significantly increased in DM(-DAPT/D1) and DM(-DAPT/D3) compared to DM, and the expressions of both *CEBPA* and *ALB* were significantly higher in DM(-DAPT/D3) over DM(-DAPT/D1). Overall, DM(-DAPT/D3) showed comparable results in terms of balanced ratios of cell types and composition. Thus, DM(-DAPT/D3) was chosen to produce mLOs for the following experiment. We also confirmed the modulation of the Notch signaling by counting cell nuclei labeled with Notch1 signals in the presence (DM) and absence [DM(-DAPT/D3)] of DAPT during the generation of mLOs (Additional file 1: Fig. S4B). The number of cell nuclei labeled with Notch signals was significantly increased by more than threefold in the absence of DAPT.

**Fig. 4:**
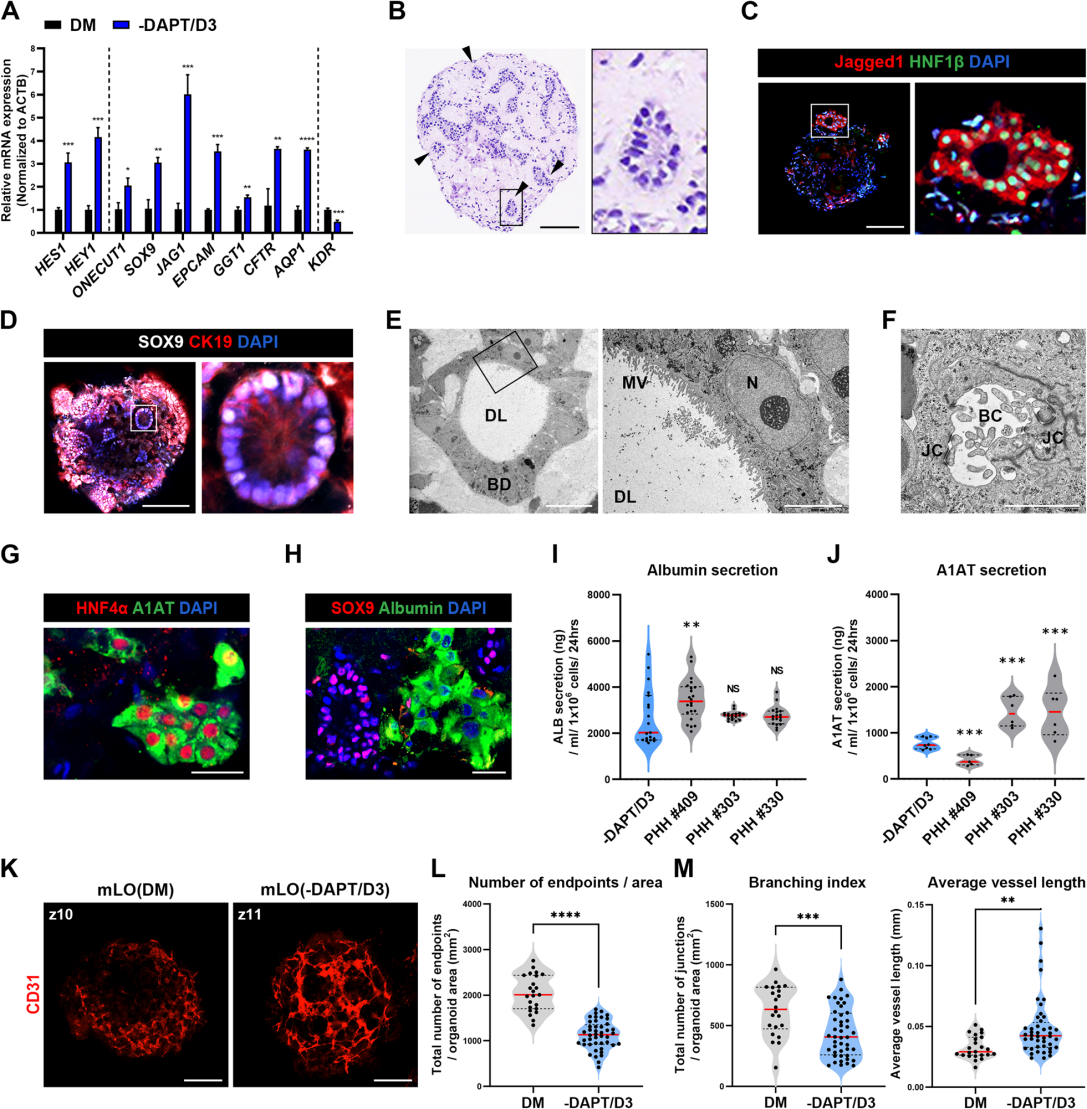
Characterization of bile ducts and endothelial networks in mLOs produced by regulating Notch activity. **A** qRT-PCR analysis of Notch target genes (*HES1* and *HEY1*), biliary markers (*ONECUT1, SOX9*, *JAG1*, *EPCAM*, *GGT1*, *CFTR,* and *AQP1*), and an endothelial tip cell marker (*KDR*) in mLOs. Data are expressed as mean ± SD (*n* = 3, normalized to *ACTB*). *p < 0.05, **p < 0.01, ***p < 0.001, and ****p < 0.0001 by unpaired *t* tests. **B** H&E staining showing multiple duct-like structures in mLO(-DAPT/D3) (arrowheads). An enlarged image of the boxed area is shown separately in the right panel. Scale bar, 200 μm. **C**, **D** Representative immunofluorescence images showing expression of Jagged 1/HNF1*β* (**C**) and SOX9/CK19 (**D**) in duct-like structures of mLO(-DAPT/D3). Scale bar, 100 μm. **E**, **F**, TEM images showing the ductal lumen (DL) surrounded by biliary ductal cell-like cells (BD) in mLO(-DAPT/D3). A high-resolution image of the boxed area in E is shown separately in the right panel. MV, microvilli; *N*, nucleus. A TEM image of bile canaliculi (BC) with junctional complexes (JC) in mLO(-DAPT/D3) is shown in F. Scale bar, 20 μm. **G**, **H** Immunofluorescence images of cells expressing both HNF4*α* and A1AT (**G**). Albumin-/SOX9 + cells and albumin + /SOX9- cells were distributed separately in a single mLO (H). Scale bar, 20 μm. **I**, **J** Albumin (I) and A1AT (J) secretions from mLO(-DAPT/D3) on day 16 compared with primary human hepatocytes (PHH) from three different donors. The secretion level was normalized to the number of cells. **p < 0.01, ***p < 0.001, and ns (not significant) compared to -DAPT/D3 (unpaired *t* tests). **K** Comparison of a single confocal *z*-section (CD31-immunofluorescence staining) between mLOs produced in DM containing DAPT throughout the whole period of differentiation [16 days] and mLOs produced in DM containing DAPT for the first 3 days followed by DM lacking DAPT for the remaining 13 days of differentiation (-DAPT/D3). Serial *z*-scan images for the two groups of mLOs at the end of differentiation (day 16) are shown in Additional file 1: Fig. S5. Scale bar, 100 μm. **L**, **M** Quantification of CD31 + endothelial networks in mLOs for discontinuous endpoints (*L*), branching and average length (M) of vessels. Confocal images of vascular structures were analyzed using AngioTool and ImageJ. Each black dot represents a single *z*-scan image (*n* ≥ 40 per group, two independent experiments). **p < 0.01, ***p < 0.001, and ****p < 0.0001 by unpaired *t* tests

We next confirmed the role of Notch signaling regulation in the formation of bile ducts and vasculature by comparing the gene expression profiles of mLO(DM) and mLO(-DAPT/D3). mLO(-DAPT/D3) showed a threefold to fourfold increase in the expression of Notch target genes *HES1* and *HEY1* compared with mLO(DM), indicating that DAPT withdrawal recovered endogenous Notch signaling (Fig. 4A). This regain in Notch activity led to significant upregulation of various genes associated with biliary formation, including *ONECUT1*, SRY-Box transcription factor 9 (*SOX9*), *JAG1*, *EPCAM*, *γ*-glutamyltransferase 1 (*GGT1*), cystic fibrosis transmembrane conductance regulator (*CFTR*), and aquaporin 1 (*AQP1*) in mLO(-DAPT/D3) (Fig. 4A). Furthermore, H&E staining showed the presence of multiple bile duct-like structures in mLO(-DAPT/D3) (Fig. 4B, arrowheads) that expressed Jagged1/HNF1*β* and SOX9/cytokeratin-19 (CK19) (Fig. 4C, D). Transmission electron microscopy (TEM) imaging also revealed bile duct-like structures with cell polarity that exhibited well-developed microvilli at the luminal surface of ducts as well as bile canaliculi together with junctional complexes (Fig. 4E, F). These bile duct-like structures were distinguished from hepatocyte-like cells (HLCs) expressing both HNF4*α* and alpha-1 antitrypsin (A1AT) (Fig. 4G). The albumin-/SOX9 + cells and SOX9-/albumin + cells were separately detected in a single mLO(-DAPT/D3) (Fig. 4H). The level of albumin secretion in mLO(-DAPT/D3) was lower than in primary human hepatocytes (PHH) from three different donors (Fig. 4I). We also measured the level of A1AT, a major endogenous protease inhibitor secreted by hepatocytes, and found that it was lower in mLOs than in PHHs, with the exception of PHHs from one donor (Fig. 4J).

Notch signaling also affects endothelial tip/stalk cell specification in the angiogenesis process [12–14]. Many studies show that Notch inhibition in angiogenesis results in more branched and abnormal vessels [63, 64]. Interestingly, mLO(-DAPT/D3) had more complex vasculatures with longer and thicker vessels and fewer branches compared with mLO(DM), in which ECs were scattered, unorganized, and displayed an excessive branching phenotype (Fig. 4K–M; Additional file 1: Fig. S5). In line with this observation, qRT-PCR analysis showed that expression of the endothelial tip cell marker *KDR* decreased by more than half in mLO(-DAPT/D3) (Fig. 4A). Interestingly, the expression of HSC-specific markers, lecithin retinol acyltransferase (*LRAT*), and protocadherin 7 (*PCDH7*) [46] was significantly increased in mLO(-DAPT/D3), compared to those in 2D-differentiated hPSC-HscLCs (Additional file 1: Fig. S6).

### Verification of luminal vasculature in liver organoids

**Fig. 5:**
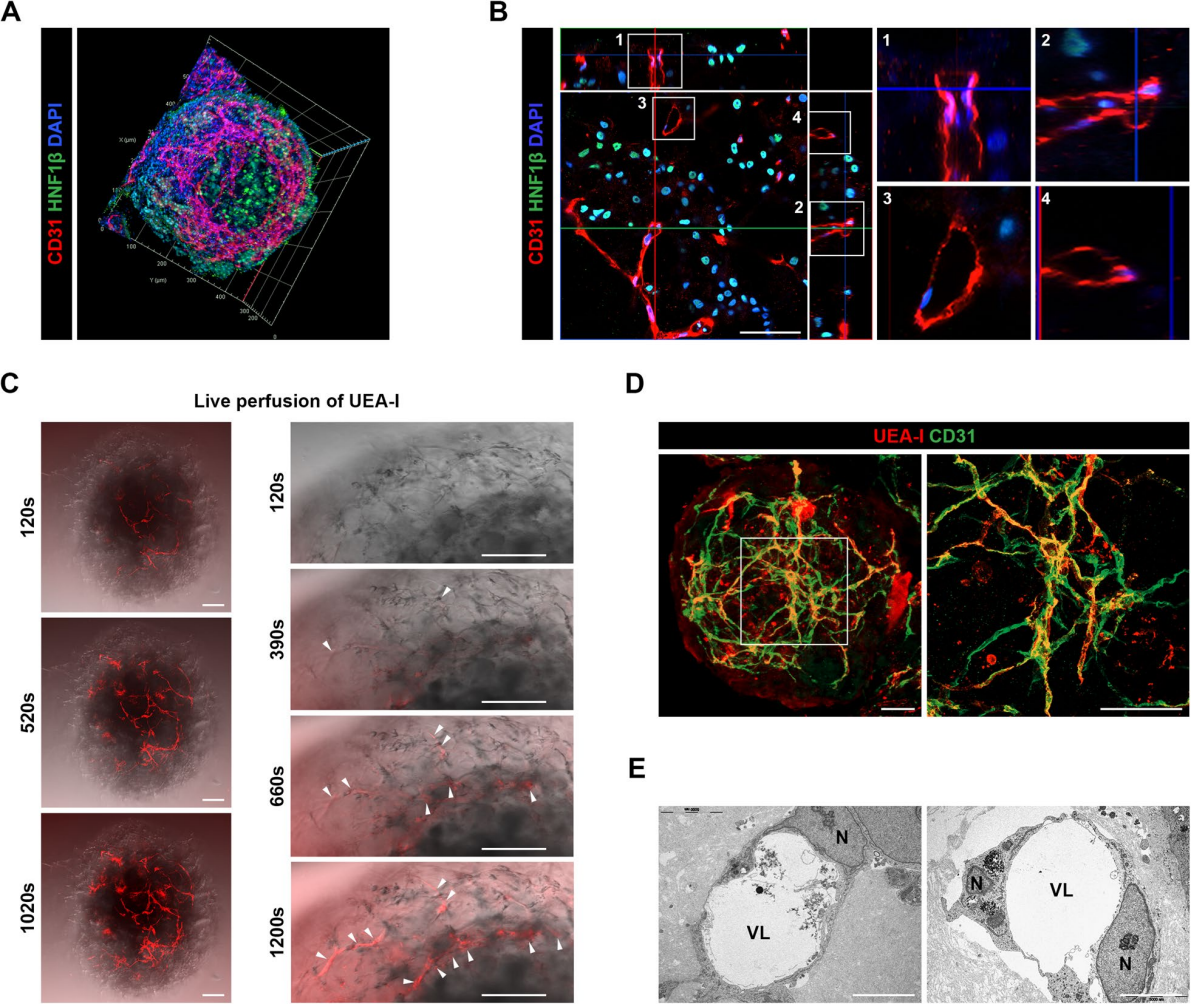
Characterization of luminal vasculature in mLO(-DAPT/D3). **A** Stereo-projection of confocal images of mLOs expressing the EC and hepatocyte markers (CD31 and HNF1*β*) on day 16 of differentiation. **B** Orthogonal projections (*x*/*y*, *x*/*z*, and *y*/*z*) of confocal *z*-stacks obtained from mLOs with vascular networks. Orthogonal views (*x*/*z* and *y*/*z*) represent planes of intersection at the position of the cross-line (x/y). Note that luminal structures (boxes #1 and #2) were created by surrounding CD31 + ECs. Two additional vascular lumens are seen in the same image (boxes #3 and #4). Scale bar, 50 μm. **C** Time-lapse confocal image sequence of mLOs after in vitro live perfusion of UEA-I-rhodamine (red). UEA-I-rhodamine was perfused into vessels in mLOs and propagated to vascular networks over time (s, seconds). High-magnification images at the periphery of the organoids are shown separately in the right panel. Arrowheads indicate rhodamine signal. Scale bar, 100 μm. **D** Immunofluorescence images of CD31 in mLOs after in vitro live perfusion of UEA-I-rhodamine. Scale bar, 100 μm. **E** TEM images showing the vascular lumens (VL) created by a single EC (left) and multiple ECs (right). *N* nucleus. Scale bars, 5 μm

In vitro formation of luminal vasculature facilitates the circulation of culture medium (i.e., nutrients and oxygen) inside organoids and may also recapitulate physiological interactions between blood vessels and parenchymal cells under pathological conditions. Numerous attempts have been made to recapitulate the human vascular microenvironment in 3D organ models in vitro by using microfluidic devices, bioprinting, and biomaterials [65]. To the best of our knowledge, however, in vitro generation of perfusable vasculature in self-organized liver organoids has not been reported. Thus, we further investigated whether the CD31 + networks produced in mLO(-DAPT/D3) generated capillary vessels with perfusable lumens. Whole-mount staining and 3D rendering of mLO(-DAPT/D3) demonstrated that CD31 + vasculature was distributed throughout whole organoids between HNF1*β* + HLCs (Fig. 5A, Additional file 2: Video 1). An orthogonal high-magnification image showed that vessels in mLOs had multiple lumens (Fig. 5B, boxed areas are separately shown in the right panels). We next attempted to directly examine the capillary lumen formation of vascular structures in mLO(-DAPT/D3) by in vitro live perfusion of rhodamine-conjugated Ulex Europaeus Agglutinin l (UEA-I-rhodamine). Notably, live time-lapse imaging of an mLO showed that UEA-I-rhodamine gradually diffused into vascular lumens over time and accumulated in vascular structures throughout the entire organoid (Fig. 5C, Additional file 3: Video 2). Whole-mount immunofluorescence staining of mLOs after in vitro live perfusion showed that UEA-I-rhodamine signals were co-labeled with CD31 + vascular structures (Fig. 5D). Electron microscopy confirmed the generation of capillary lumens produced by one or two ECs in the organoid (Fig. 5E).

### Responses of mLOs to fibrosis-related cytokines

**Fig. 6:**
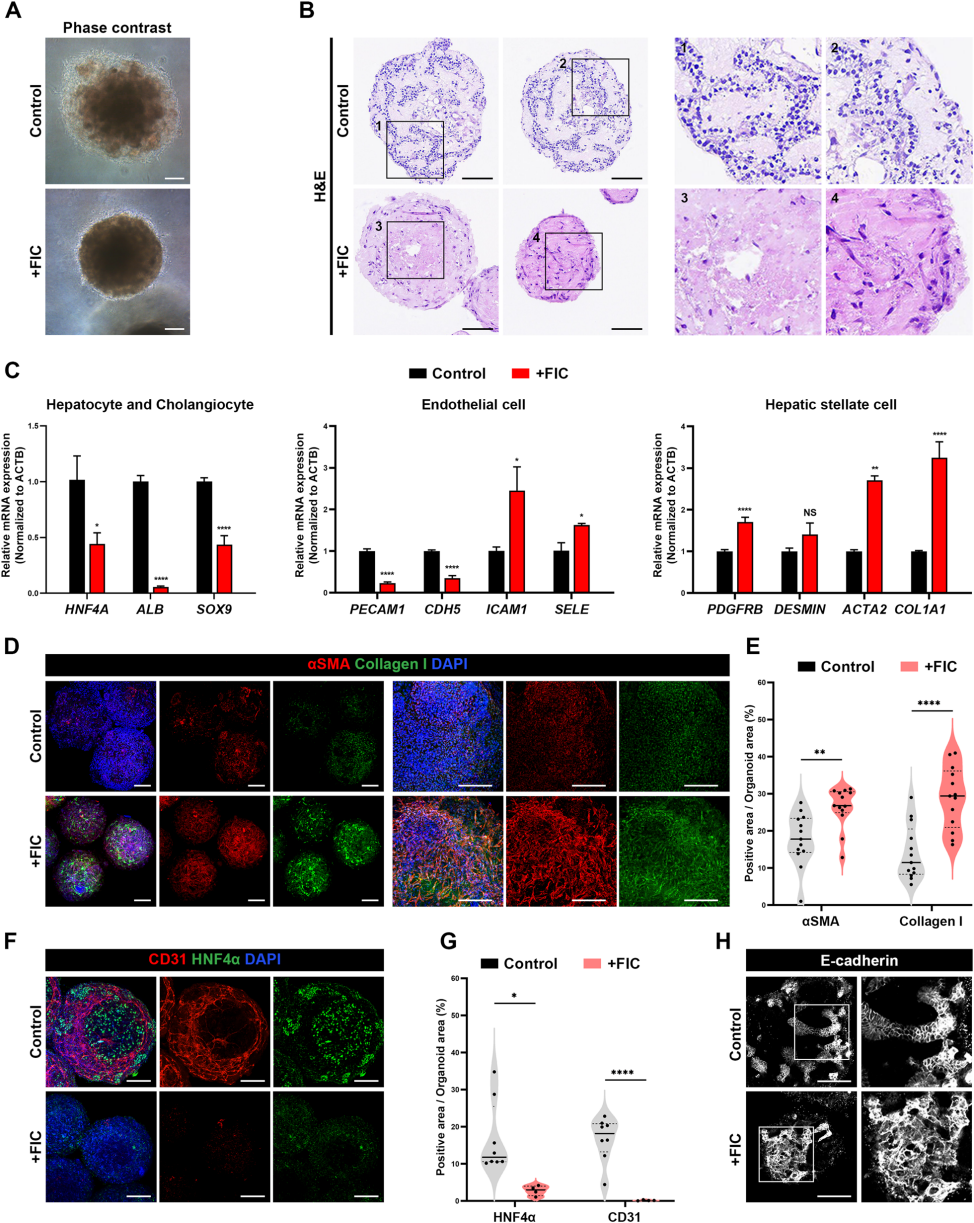
Exposure of mLO(-DAPT/D3) to fibrosis-associated cytokines. **A** Phase-contrast images after treatments of mLOs with (+ FIC) or without (Control) fibrosis-inducing cytokine cocktail (FIC, a mixture of TGF*β*1, TNF*α*, IL-6, and IL-1*β*) for 5 days. Scale bar, 100 μm. **B** H&E staining showing a change in tissue architecture in mLOs after treatment with FIC. Note that cell platelike epithelial structures were destroyed by exposure to FIC. Scale bar, 100 μm. **C**, qRT-PCR analysis of changes in gene expression associated with hepatic epithelium (*HNF4A*, *ALB,* and *SOX9*), endothelium (*PECAM1*, *CDH5, ICAM1*, and *SELE*), and HSCs (*PDGFRB*, *DESMIN*, *ACTA2*, and *COL1A1*). Data are expressed as mean ± SD (n = 3, normalized to *ACTB*). *p < 0.05, **p < 0.01, ***p < 0.001, ****p < 0.0001, and NS (not significant) by unpaired *t* tests. **D** Whole-mount immunofluorescence images of mLOs for the expression of human collagen I and *α*-SMA, which are key markers of HSC activation. Scale bar, 200 μm. **E** Quantification of immunoreactive signals for human collagen I and *α*-SMA. Each black dot represents the percent positive area of immunoreactive signal in each mLO (*n* = 13 per group, **p < 0.01 and ****p < 0.0001 by unpaired *t* test). **F** Whole-mount immunofluorescence images of EC and hepatocyte marker (CD31 and HNF1*β*) expression in mLOs. Scale bar, 200 μm. **G** Quantification of immunoreactive signals for HNF4*α* and CD31. Each black dot represents the percent positive area of the immunoreactive signal in each mLO (*n* ≥ 4 per group, *p < 0.05 and **p < 0.0001 by unpaired *t* tests. **H** Single *z*-scan immunofluorescence images of E-cadherin expression in control and FIC-treated mLOs. Scale bar, 100 μm

The potential application of mLO(-DAPT/D3) for the modeling of liver fibrosis was then examined after sustained exposure to a fibrosis-inducing cytokine cocktail (FIC, a mixture of TGF*β*1, TNF*α*, IL-6, and IL-1*β*). Treatments of mLOs to FIC for 5 days resulted in the shrinkage of mLOs (Fig. 6A). Histological analysis of mLOs demonstrated a substantial decrease in the epithelial cell population and disappearance of cell platelike and duct-like structures together with an increased proportion of ECM area (Fig. 6B, boxed areas are shown separately in the right panels). Typical marker genes for hepatocytes (*HNF4A*, *ALB*), cholangiocytes (*SOX9*), and ECs (*PECAM1*, *CDH5*) were significantly decreased in mLOs exposed to FIC (Fig. 6C). Notably, in response to FIC, the expression of activation markers for ECs (*ICAM1 and SELE*) and HSCs (*ACTA2* and *COL1A1*) significantly increased, together with upregulation of other HSC marker genes, *PDGFRB* and *DESMIN* (Fig. 6C). Whole-mount staining images showed that FIC substantially increased the positive area for fibrotic markers collagen I and *α*-SMA (Fig. 6D, E), suggesting that HscLCs in mLOs were activated by cytokine exposure. In addition, CD31 + and HNF1*β* + areas decreased in response to FIC, representing damage to hepatocytes and ECs in mLOs (Fig. 6F, G). Furthermore, mLOs treated with FIC showed an appearance of fibroblastic cells (Fig. 6B) and aberrant pattern of E-cadherin expression (Fig. 6H), suggesting induction of events associated with epithelial-to-mesenchymal transition [62]. We also compared the response of mLOs assembled with different combinations of cell types (HE + EC + HscLC, HE + HscLC, and HE only) to FIC (Additional file 1: Fig. S7). Overall, the highest upregulation of different genes related to the early fibrotic events (*ACTA2*, *COL1A1*, and *TGFB1*) and inflammatory cytokines (*TNFA*, *IL-6*, and *IL-8*) was observed in mLOs consisting of all three cell types.

## Discussion

Although recent studies demonstrate the connection of endothelial networks in mLO transplants to host vessels [22, 23, 28], in vitro formation of perfusable luminal vasculature in self-organized mLOs has not been reported. We previously showed that hiPSC-derived mLOs can be produced by assembling HE, mesenchymal stem cells, and ECs [36]. We found that mLOs generated EC networks that secrete FVIII, correcting the bleeding phenotype of HA [36], but whether the ECs created luminal vessels was not investigated. In the present study, we reproducibly generated vascularized mLOs and assessed the perfusability of vascular lumens in vitro.

Previous studies reported that the presence of mesodermal progenitor [66–68] or pericytes [54, 55] is critical for vasculature formation in various organoids. In agreement with these findings, we found that hPSC-derived HscLCs were crucial to the development of functional microvasculature in mLOs. We also confirmed the presence of luminal tubular networks of vasculature by live-cell imaging, showing that the microvessels of mLOs were gradually perfused with a culture medium containing rhodamine-UEA-I in vitro. A series of confocal *z*-scans of mLOs revealed that the vessels were well distributed inside mLOs. TEM analysis supported these findings by revealing luminal vascular structures with cell polarity produced by ECs. A recent study generated vascular organoids consisting of ECs and pericytes from hPSCs and showed that a close association between ECs and pericytes is required to generate endothelial tubes in vitro [54, 55]. Therefore, the previous and present studies suggest that the incorporation of cells displaying features of pericytes is required for promoting the in vitro formation of functional vasculature in mLOs.

We found that the inclusion of HscLCs and subsequent vascularization substantially inhibited apoptotic cell death and promoted the growth of mLOs in vitro. Technically, in vitro formation of mLOs with functional vasculature facilitate the delivery of nutrients, oxygen, and growth factors from culture medium into the core of organoids. In particular, recent organoid studies have begun to use assembloids that are produced by combining multiple organoids, each resembling different tissues, to obtain a better understanding of the crosstalk between different tissues in pathological conditions [69–74]. Thus, generating mLOs with functional vessels may allow the formation of relatively large organoids or assembloids that more closely mimic the complexity of tissue architecture without necrotic cores. Therefore, in vitro generation of luminal vascular structures is crucial for producing usable mLOs from both biological and technical aspects.

To the best of our knowledge, there are no studies showing the generation of both functional vasculature and bile ducts in individual mLOs. The asymmetric activation of signaling pathways by paracrine cytokines and cell–cell interaction is required for the differentiation of diverse cell lineages and typical tissue morphogenesis during normal development processes [12, 13, 16, 75–78]. Notch signaling is activated by cell–cell direct interactions and plays an important role in both biliary ductal formation and vascular development [12–14, 16, 17]. During bile duct formation, portal fibroblasts that express the Notch ligand Jagged1 transduce Notch signaling and induce preferential differentiation of nearby bipotent hepatoblasts into cholangiocytes rather than hepatocytes [16, 17]. The Notch pathway also mediates endothelial cell–cell interactions and contributes to functional vessel patterning and outgrowth. Endothelial tip cells expressing another Notch ligand, Delta-like 1, activate Notch on ECs to laterally inhibit neighboring cells from becoming tip cells, implying that Notch signaling is important for vascular outgrowth by preventing the excessive branching of vessels [12, 15, 63, 64]. The DM that is widely used to differentiate mLOs contains DAPT, a Notch signaling inhibitor, to enrich the parenchymal hepatocyte population by inhibiting biliary differentiation in organoids [37–39]. On the other hand, proper activation of Notch signaling is necessary for the generation of bile ducts in cholangiocyte organoids [58, 59]. In our study, we attempted to modulate the duration of inhibition and activation of endogenous Notch signaling in mLOs by removing DAPT in the DM at different time points across 16 days of differentiation. We found that inhibition of endogenous Notch signaling by DAPT for an initial 3–5 days followed by a release of inhibition using DM lacking DAPT for the remainder of the differentiation period [11–13 days] increased Jagged1 expression and led to the production of multiple biliary ducts expressing Jagged1, HNF1*β*, SOX9, and CK19 in mLOs. Furthermore, in the resulting mLOs, the number of endpoints and branching index of vasculature decreased, whereas average vessel length increased, suggesting that the outgrowth of blood vessels was promoted by optimizing Notch activity. Therefore, our observation strongly suggests that the exact time window for the modulation of Notch signaling is essential for the generation of both bile ducts and vasculature in mLOs. The molecular mechanism underlying the Notch-mediated fate choices and differentiation of hepatocytes, biliary cells, and ECs in individual mLOs remains to be investigated. Further studies using single-cell RNA sequencing and functional network analysis would allow a better understanding of the concurrent differentiation and functional association of multiple cell types in mLOs.

A limitation of our study is the absence of immune cells in mLOs. Kupffer cells are liver-resident macrophages that secrete a variety of inflammatory cytokines and play critical roles in the pathogenesis of liver diseases [79]. There are no reports demonstrating the successful derivation of Kupffer cells from iPSCs, probably due to controversial issues regarding their embryonic origin [80]. A recent report shows that iPSCs can be differentiated into functional Kupffer cells that display multiple phenotypes of human primary Kupffer cells by exposing iPSC-derived macrophage precursors to hepatic cues [81]. This report also demonstrates that iPSC-derived Kupffer cells are applicable to the modeling of liver diseases and drug toxicity testing. Therefore, we expect that the inclusion of iPSC-derived Kupffer cells in the mLOs produced in our study would allow the generation of organoids containing all major parenchymal and non-parenchymal cells, which more precisely reflects inflammation-associated pathogenic events in acute and chronic liver diseases. Nonetheless, a pro-fibrotic cytokine cocktail appears to drive fibrotic events in mLOs produced in our study, as evidenced by the upregulation of fibrotic markers (collagen I and *α*-SMA) and an EC activation marker (ICAM) together with the destruction of tissue architecture and cell death in the organoids. In light of our observation, incorporating Kupffer cells in our mLOs is a pressing priority for accurately modeling chronic diseases that cause liver fibrosis, such as non-alcoholic steatohepatitis and Wilson’s disease.

Although we previously showed that HLCs can be generated from hPSCs [41, 82], mesenchymal stem cells [83], and directly reprogrammed fibroblasts [84], stem cell-derived HLCs exhibited fetal phenotypes and showed low drug-metabolizing activity. We also previously demonstrated that the generation of 3D HLC spheroids from hPSCs increased drug-metabolizing activity but at a level lower than that of human primary hepatocytes [34, 35]. In the present study, we found that the capacity of mLOs to secrete albumin and A1AT is still lower than PHHs. Therefore, enhancing the hepatic functions of parenchymal HLCs is urgently needed for applying mLOs in drug-screening and regenerative medicine. Recent studies reported that 3D bioprinting of hiPSC-derived HLC spheroids with ECs and MSCs resulted in enhanced expression of drug-metabolizing enzymes [85]. Furthermore, other studies showed that microfluidic devices and tissue-derived ECM promoted structural and functional maturation of organoids that recapitulate key features of the liver [26, 32, 86–90] and other organs [91, 92]. Thus, utilizing microfluidic chips and tissue-specific ECM could be a breakthrough to achieve the functional maturation of mLOs. Nevertheless, our studies suggest the feasibility of using mLOs containing multiple non-parenchymal cells to model liver diseases. Indisputably, non-parenchymal cells create a complex hepatic niche for retaining normal liver function and contribute to the initiation, progression, and manifestation of liver diseases. Therefore, the incorporation of Kupffer cells into mLOs with enhanced drug-metabolizing activity may pave the way toward precise in vitro modeling of liver diseases and innovative drug-screening systems.

Concurrent differentiation of the various cell types under a single culture condition may differentially affect the survival or proliferation of each cell type in organoids. Given that hepatic endodermal cells are highly proliferative, and DM is the medium previously designed for the growth and differentiation of hepatic progenitors, we reduced the assembling ratio of HE in mLOs and increased the relative proportion of ECs to support the luminal vascularization of organoids. However, the hepatic parenchymal cells occupy the main part of the normal liver and play a central role in a wide range of hepatic functions. Therefore, the assembly ratio of multiple cell types should be carefully adjusted based on culture conditions to produce functional mLOs.

## Conclusion

In conclusion, we developed mLOs with vasculature and bile ducts by combining HE, ECs, and HscLCs derived entirely from hPSCs. Our findings indicate that HscLCs play a crucial role in vessel formation in mLOs. Furthermore, we found that precise control of Notch activity is required for both bile duct development and vascular outgrowth in mLOs. Our mLOs generated perfusable microvessels with vascular lumens and showed early fibrotic events in response to pro-fibrotic cytokines at cellular and molecular levels. These hPSC-derived mLOs are a promising tool for the modeling of liver diseases mediated by parenchymal and non-parenchymal interplay.

## Supplementary Information

**Additional file 1**. Supplementary tables and figures.

**Additional file 2**. **Video 1.** 3D rotation of stereo-projection of mLOs expressing the EC and hepatocyte makers

**Additional file 3**. **Video 2.** Live perfusion of luminal vasculature in mLO(-DAPT/D3).

## Data Availability

All data generated or analyzed during this study are included in this published article and its supplementary information files. Additional experimental details and more detailed data used or analyzed during the current study are available from the corresponding author upon reasonable request.

## Abbreviations

mLOs: Multilineage liver organoids
HscLCs: Hepatic stellate cell-like cells
ECs: Endothelial cells
HSCs: Hepatic stellate cells
ECM: Extracellular matrix
hPSCs: Human pluripotent stem cells
hiPSC: Human induced pluripotent stem cell
HE: Hepatic endoderm
DM: Differentiation medium
qRT-PCR: Quantitative reverse-transcription polymerase chain reaction
GAPDH: Glyceraldehyde 3-phosphate dehydrogenase
ACTB: Beta-actin
FOXA2: Forkhead box A2
SOX17: SRY-box 17
HNF4*α*: Hepatocyte nuclear factor 4-alpha
HNF1*β*: Hepatocyte nuclear factor 1-beta
AFP: Alpha-fetoprotein
EPCAM: Epithelial cell adhesion molecule
MACS: Magnetic-activated cell sorting
VECAD: Vascular endothelial cadherin
vWF: Von Willebrand factor
PDGFR*β*: Platelet-derived growth factor receptor-beta
*α*-SMA: Alpha-smooth muscle actin
GFAP: Glial fibrillary acidic protein
EdU: 5-Ehynyl-2′-deoxyuridine
SOX9: SRY-box transcription factor 9
GGT1: Glutamyltransferase 1
CFTR: Cystic fibrosis transmembrane conductance regulator
AQP1: Aquaporin 1
CK19: Cytokeratin 19
HLCs: Hepatocyte-like cells
A1AT: Alpha-1 antitrypsin
PHH: Primary human hepatocyte
LRAT: Lecithin retinol acyltransferase
PCDH7: Protocadherin 7
UEA-I-rhodamine: Rhodamine-conjugated Ulex Europaeus Agglutinin I
FIC: Fibrosis-inducing cytokine cocktail
TGF*β*1: Transforming growth factor beta 1
TNF*α*: Tumor necrosis factor alpha
IL-6: Interleukin-6
IL-1*β*: Interleukin-1 beta
CEBPA: CCAAT enhancer-binding protein alpha
ALB: Albumin
LRAT: Lecithin retinol acyltransferase
PCDH7: Protocadherin 7
IL-8: Interleukin-8
