# Supplementary material for: Generation of multilineage liver organoids with luminal vasculature and bile ducts from human pluripotent stem cells via modulation of Notch signaling
Source: Stem Cell Res Ther. 2023 Feb 3;14:19. doi: 10.1186/s13287-023-03235-5 (PMC9898924; doi:10.1186/s13287-023-03235-5)
Supplement: Supplementary file 1 — Additional file 1. Supplementary tables and figures. [file 13287_2023_3235_MOESM1_ESM.docx]

**Additional file 1: Supporting information**

**Generation of multilineage liver organoids with luminal vasculature and bile ducts from human pluripotent stem cells via modulation of Notch signaling**

Hyo Jin Kim^1^, Gyeongmin Kim^1^, Kyun Yoo Chi^1^, Hyemin Kim^2^, Yu Jin Jang^3^, Seongyea Jo^1,2^, Jihun Lee^1^, Youngseok Lee^1^, Dong-Hun Woo^4^, Choongseong Han^4^, Sang Kyum Kim^5^, Han-Jin Park^2^, Jong-Hoon Kim^1^

^1^Laboratory of Stem Cells and Tissue Regeneration, Department of Biotechnology, College of Life Sciences and Biotechnology, Korea University, Seoul, 02841, South Korea

^2^Department of Predictive Toxicology, Korea Institute of Toxicology, Daejeon, 34114, South Korea

^3^Department of Molecular Biosciences, The University of Texas at Austin, Austin, Texas 78712, USA

^4^Department of Stem cell Biology, NEXEL Co., Ltd, Seoul, 07802, South Korea

^5^College of Pharmacy, Chungnam National University, Daejeon, 34134, South Korea

**Correspondence: Jong-Hoon Kim**

e-mail: [jhkim@korea.ac.kr](mailto:jhkim@korea.ac.kr)

**List of Supporting information**

- **Expanded Materials and Methods**
- **Supplementary tables**

**Table S1.** Antibodies used for immunocytochemistry.

**Table S2.** Primers used for qRT-PCR.

- **Supplementary Figures**

**Fig. S1.** Flow cytometry analyses of EPCAM, AFP, and CD31 in hPSC-derived HE and ECs.

**Fig. S2.** Reactivity of hPSC-derived HscLCs to TGFβ1 treatment.

**Fig. S3.** Comparison of mLOs assembled in different cell ratios for vascularization.

**Fig. S4.** Comparison of hepatocyte- and bile duct-related gene expression pattern in mLOs after Notch modulation.

**Fig.** **S5.** Serial z-scan confocal images of CD31+ vasculature in mLOs

**Fig. S6.** Expression of HSC marker genes in hPSC-derived HscLCs and mLO(-DAPT/D3).

**Figure S7.** Responses of mLOs assembled in different combinations of cell types to FIC.

- **Supplementary Video 1**. 3D rotation of stereo-projection of mLOs expressing the EC and hepatocyte makers.
- **Supplementary Video 2.** *In vitro* Live perfusion of luminal vasculature in mLO(-DAPT/D3).

**Expanded Materials and Methods**

**Generation of hPSC-derived HE**

All experimental procedures were approved by the Institutional Review Board of Korea University, Seoul, Korea (IRB nos. 2019-0334-04 and 2020-0319-03). The human embryonic stem cell (hESC) line BG01 (WiCell Research Institute, Madison, WI, USA) was cultured and passaged on dishes coated with Matrigel (M354277, Corning) using mTeSR1 medium (5850, Stemcell Technologies). Hepatic differentiation of hPSCs was induced as previously described and summarized in Fig. 1A^1-5^. Briefly, BG01 hESCs were differentiated into endodermal cells in RPMI-1640 media (31800-022, Gibco) supplemented with 100 ng/ml activin A (338-AC, R&D Systems) and 2 μM CHIR99021 (4423, Tocris Bioscience) for 1.5 days. Cells were further differentiated into endodermal cells in RPMI-1640 media (31800-022, Gibco) in the presence of 100 ng/ml activin A (338-AC, R&D Systems) and 1 mM sodium butyrate (B5887, Sigma-Aldrich) for an additional 1.5 days. To induce HE, endodermal cells were grown in RPMI-1640 media (31800-022, Gibco) containing 1× B27 supplement (17504-044, Gibco), 20 ng/ml bone morphogenetic protein 2 (BMP2, 120-02, R&D Systems), and 30 ng/ml fibroblast growth factor 4 (FGF4, 235-F4, R&D Systems) for 2 days.

**Generation and purification of hPSC-derived ECs and HscLCs**

BG01 hESCs were differentiated into ECs as previously described with minor modifications^4,5^ (Fig. 1C). Briefly, BG01 hESCs were differentiated into mesodermal cells for 2 days in dishes coated with 5 μg/ml fibronectin (FC010, Millipore) containing DMEM/F12 (12500-062, Gibco) supplemented with 200 μM ascorbic acid (A4544, Sigma), 1% ITS-X (51500-056, Gibco), 1% penicillin/streptomycin (15140-122, Gibco), 3 μM CHIR99021 (4423, Tocris Bioscience), 10 μM Y-27632 (1254, Tocris Bioscience), and 2 ng/ml activin A (338-AC, R&D Systems). Resulting mesodermal cells were further differentiated into vascular progenitor cells for an additional 4 days in Stemline II (S1092, Sigma) supplemented with 50 ng/ml VEGF165 (100-20, Peprotech) and 1% penicillin/streptomycin (15140-122, Gibco). On day 6 of differentiation, vascular progenitor cells were dissociated into single-cell suspension using TrypLE (12604-021, Gibco) and passed through a 40-μm cell strainer (352340, BD Biosciences). After centrifugation, collected cells were washed in magnetic-activated cell sorting (MACS) buffer (0.1% BSA in PBS) and incubated with mouse anti-human CD31 antibody (555444, BD Biosciences) for 30 min. After centrifugation, cells were washed with MACS buffer and incubated with anti-mouse IgG microbeads (130-048-401, Miltenyi Biotech) for 20 min. After washing, CD31+ cells were separated using a MACS LS column (130-042-401, Miltenyi Biotech). CD31+ ECs were replated in 5 μg/ml fibronectin-coated dishes and cultured in Stemline II media supplemented with penicillin/streptomycin and 50 μg/ml VEGF165. CD31- cells were further differentiated into HscLCs in plates coated with 3 μg/ml fibronectin and 10 μg/ml collagen I (C7661, Sigma) using the pericyte medium (1201, ScienCell) supplemented with pericyte growth supplement, 5% FBS, 10 ng/ml PDGF-BB (100-14b, Peprotech), 10 ng/ml bFGF (100-18b, Peprotech), and 5 μM retinol (R7632, Sigma)^6-8^. To examine the reactivity to pro-inflammatory cytokine, CD31- cell-derived HscLCs were cultured in the presence or absence of 10 ng/ml TGFβ1 (240-B, R&D Systems) for 2 days in the pericyte medium supplemented with pericyte growth supplement, 0.5% FBS, 10 ng/ml PDGF-BB, 10 ng/ml bFGF.

**Fibrotic induction of mLOs**

For modeling liver fibrosis, mLOs were treated with a fibrosis-inducing cocktail [FIC, a mixture of 10 ng/ml TGFβ1 (240-B, R&D Systems), 10 ng/ml TNFα (AF-300-01A, Peprotech), 10 ng/ml IL-6, and 10 ng/ml IL-1β (AF-200-01B, Peprotech)] for 5 days in DM without DAPT and A83-01. The DM containing FIC was refreshed every 3 days.

**Primary human hepatocyte culture**

Cryopreserved primary human hepatocytes from three different donors (454543, Corning; lot #409, #303, and #330) were thawed using a Corning Gentest High Viability CryoHepatocyte recovery kit and plated on 20 μg/ml collagen I-coated 24-well plates in Corning Gentest CryoHepatocyte plating medium according to the instructor’s guide. Four hours after cell plating, the culture medium was changed to Corning Hepatocyte culture medium. After 24 hours, the culture medium was collected, centrifuged 2000rpm for 20 min at 4℃ to remove debris, and used to analyze albumin and A1AT secretion.

**Albumin and A1AT secretions**

After 24 h in culture, the culture media of mLOs or PHH were harvested and centrifuged to remove cell debris. Albumin secretion in the supernatant was measured using a human albumin enzyme-linked immunosorbent assay kit (E88-129, Bethyl Laboratories) and human Alpha-1-Antitrypsin ELISA kit (E88-122, Bethyl Laboratories) according to the manufacturer’s instructions. Absorbance was measured at 450 nm using a SpectraMAX i3x reader (Molecular Devices). All data were normalized by the number of cells.

**Flow cytometry**

BG01-derived HscLCs were dissociated using TrypLE and filtered through 40-μm cell strainers (352340, BD). Cells were incubated with primary antibodies followed by secondary antibodies for 30 min each at 4°C. Between each step, dissociated cells were rinsed in FACS buffer (0.1% BSA in PBS). Labeled cells were analyzed using a BD Accuri C6 Flow Cytometer System (BD Biosciences) with Accuri C6 and FlowJo software (Tree Star Inc). Primary and secondary antibodies are listed in [Additional file 1: Supplementary Table 1].

**Histologic analysis of paraffin-embedded organoid sections**

Organoids were washed three times with cold PBS and fixed in 4% paraformaldehyde solution for 1 h at room temperature on a rocker. The fixed organoids were rinsed with PBS three times, dehydrated, and embedded in paraffin blocks, and sectioned at 5 μm. After deparaffinization and rehydration of organoid sections, hematoxylin and eosin (H&E) staining was performed.

**Transmission electron microscopy**

mLOs were fixed in 3% glutaraldehyde overnight at 4°C, washed in 0.1 mol/L sodium cacodylate buffer, and incubated for 1 h in 4% osmium tetroxide. mLOs were then washed, dehydrated in an ethanol series, and embedded in propylene oxide/LX112. Specimens were then sectioned and stained with 2% uranyl acetate followed by lead citrate. Images were obtained using a Hitachi transmission electron microscope (TEM).

**5-ethynyl-2’-deoxyuridine (EdU) incorporation assay**

For EdU incorporation assay, organoids were incubated with 10 μM EdU for 6 h. Cells were washed three times with PBS, fixed with 4% paraformaldehyde, and permeabilized with 0.5% Triton-X100 for 20 min. After washing, EdU incorporation was accessed using a Click-it EdU Alexa Fluor 488 imaging kit (C10337, Thermo Fisher Scientific) according to the manufacturer’s instructions. Whole-mount immunostaining and imaging of labeled organoids were then performed as described above. Image quantification was performed using ImageJ.

**Supplementary Tables**

**Table S1. Primers used for qRT-PCR.**

| **Gene** | **Forward primer sequence** | **Reverse primer sequence** |
| --- | --- | --- |
| *GAPDH* | AGGGCTGCTTTTAACTCTGGT | CCCCACTTGATTTTGGAGGGA |
| *ACTB* | AGAGCTACGAGCTGCCTGAC | AGC ACT GTG TTG GCG TAC AG |
| *POU5F1* | AGTGAGAGGCAACCTGGAGA | ACACTCGGACCACATCCT TC |
| *T* | TGCTTCCCTGAGACCCAGTT | GATCACTTCTTTCCTTTGCATCAAG |
| *MIXL1* | CTGGGAGAGACACATCAGCA | AGGCTTGGAGAGAACACAGG |
| *PECAM1* | CCTGATGCCGTGGAAAGC | TCCAGGGATGTGCATCTG |
| *PDGFRB* | CCCTTATCATCCTCATCATGC | CCTTCCATCGGATCTCGTAA |
| *SOX17* | GTGTGAATCTCCCCGACAG | GCAACAACAAAAACCCAGGA |
| *FOXA2* | TTCTCCATCAACAACCTCATGTCC | GTAGTGCATCACCTGTTCGTAGG |
| *HNF1B* | GCCCAGTTTCCCTTCTATGC | CGGCTTTCTTGCTTCCTCTT |
| *CDH5* | GCCATCGATAATTCTGGACG | CTTCCACCACGATCTCATAC |
| *DESMIN* | CTGAGCAAAGGGGTTCTGAG | ACTTCATGCTGCTGCTGTGT |
| *CSPG4* | CTTGCTGTGGCTGTGCCTTTT | GGAACTGTGTGACCTGGAAGA |
| *ACTA2* | CCGACCGAATGCAGAAGGA | ACAGAGTATTTGCGCTCCGGA |
| *HEY1* | CTTTCGGCTCCTTCCACTTA | TTCCCCTCCCTCATTCTACA |
| *HES1* | CTACCCCAGCCAGTGTCAA | GAATGTCCGCCTTCTCCAG |
| *ONECUT1* | CCCAAACCCTGGAGCAAACT | GGGTGTGTTGCCTCTATCCT |
| *SOX9* | CAGCGAACGCACATCAAGAC | GTTCTGGTGGTCGGTGTAGT |
| *JAG1* | CTCAACGGGGGAACTTGTAG | TGGGGAACACTCACACTCAA |
| *EPCAM* | AATCGTCAATGCCAGTGTACTT | TCTCATCGCAGTCAGGATCATAA |
| *GGT1* | TCCTGGTGCTGGTCATTGT | GTGCTGTTGTAGATGGTGAGG |
| *CFTR* | AGTCCTTGCCCTTTTTCAGG | GCCTTCCGAGTCAGTTTCAG |
| *AQP1* | AATGACCTGGCTGATGGTGT | GGTGTCCAAGGGCTACAGAG |
| *KDR* | CAGACGGACAGTGGTATGG | AGTGATATCCGGACTGGTAG |
| *HNF4A* | AAGAGGAACCAGTGCCGCTA | CGCATTGATGGAGGGCAG |
| *ALB* | GCCTGCTGACTTGCCTTCATTAG | TCAGCAGCAGCACGACAGAGTA |
| *ICAM1* | GGAAATACTGAAACTTGCTGCCTAT | ACACATGTCTATGGAGGGCCAC |
| *SELE* | GGCAGTGGACACAGCAAATC | TGGACAGCATCGCATCTCA |
| *COL1A1* | GACACAGAGGTTTCAGTGG | CACCCTTAGCACCAACAG |
| *NESTIN* | GAAACAGCCATAGAGGGCAAA | TGGTTTTCCAGAGTCTTCAGTGA |
| *CEBPA* | GCAAACTCACCGCTCCAAT | TTAGGTTCCAAGCCCCAAGT |
| *TGFB1* | CGAGCCTGAGGCCGACTA C | AGATTTCGTTGTGGGTTTCCA |
| *TNFA* | CCTCTCTCTAATCAGCCCTCTG | GAGGACCTGGGAGTAGATGAG |
| *IL-6* | GGAGACTTGCCTGGTGAAAA | GCATTTGTGGTTGGGTCAG |
| *IL-8* | CAGCCTTCCTGATTTCTGC | AGTTTTCCTTGGGGTCCAG |

**Table S2. Antibodies used for immunocytochemistry.**

| **Antibody target** | **Supplier** | **Catalog number** | **Dilution** |
| --- | --- | --- | --- |
| anti-FoxA2 antibody | Milipore | 07-633 | IF (1:200) |
| anti-Sox17 antibody | R&D Systems | AF1924 | IF (1:200) |
| anti-HNF4α antibody | Cell signaling | 3113 | IF (1:500) |
| anti-HNF1β antibody | R&D Systems | AF3330 | IF (1:100) |
| anti-CD31 antibody | Bethyl laboratories | IHC00055 | IF (1:100) |
| anti-CD31 antibody | BD Biosciences | BD555444 | IF (1:100), FACS (1:200) |
| anti-PDGFRβ antibody | Cell signaling | 3169 | IF (1:100), FACS (1:200) |
| anti-VE-cadherin antibody | Santa Cruz | sc52751 | IF (1:50) |
| anti-vWF antibody | Milipore | AB7356 | IF (1:400) |
| anti-αSMA antibody | Sigma | A2547 | IF (1:200) |
| anti-GFAP antibody | Dako | Z0334 | IF (1:200) |
| anti-Nestin antibody | R&D Systems | MAB1259 | IF (1:200) |
| anti-NG2 antibody | BD Biosciences | BD554275 | IF (1:100) |
| anti-NG2 antibody | Milipore | AB5320 | IF (1:200), FACS (1:400) |
| anti-Cleaved caspase 3 antibody | Cell signaling | 9664 | IF (1:200) |
| anti-E-cadherin antibody | R&D Systems | AF648 | IF (1:200) |
| anti-Jagged 1 antibody | Cell signaling | 70109 | IF (1:200) |
| anti-Notch1 antibody | Santa Cruz | sc376403 | IF (1:200) |
| anti-Sox9 antibody | Milipore | AB5535 | IF (1:200) |
| anti-CK19 antibody | Dako | M0888 | IF (1:200) |
| anti-alpha 1 anti-trypsin antibody | GeneTex | GTX77515 | IF (1:200) |
| anti-Albumin antibody | Bethyl laboratories | A80-129A | IF (1:100) |
| anti-Collagen I antibody | Milipore | 234167 | IF (1:200) |
| anti-EPCAM antibody | Cell signaling | 2929 | FACS (1:400) |
| anti-AFP antibody | Dako | A0001 | FACS (1:400) |
| Normal mouse IgG | Milipore | 12-371 | FACS (1:400) |
| Normal rabbit IgG | Milipore | 12-370 | FACS (1:400) |
| Donkey anti-mouse IgG Alexa 647 | Invitrogen | A31571 | IF (1:400), FACS (1:1000) |
| Donkey anti-mouse IgG Alexa 568 | Invitrogen | A10037 | IF (1:400), FACS (1:1000) |
| Donkey anti-mouse IgG Alexa 488 | Invitrogen | A21202 | IF (1:400), FACS (1:1000) |
| Donkey anti-mouse IgG Alexa 594 | Invitrogen | A21203 | IF (1:400), FACS (1:1000) |
| Donkey anti-rabbit IgG Alexa 647 | Invitrogen | A31573 | IF (1:400), FACS (1:1000) |
| Donkey anti-rabbit IgG Alexa 568 | Invitrogen | A10042 | IF (1:400), FACS (1:1000) |
| Donkey anti-rabbit IgG Alexa 488 | Invitrogen | A21206 | IF (1:400), FACS (1:1000) |
| Donkey anti-rabbit IgG Alexa 594 | Invitrogen | A21207 | IF (1:400), FACS (1:1000) |
| Donkey anti-goat IgG Alexa 488 | Invitrogen | A11055 | IF (1:400), FACS (1:1000) |

**Supplementary Figures**


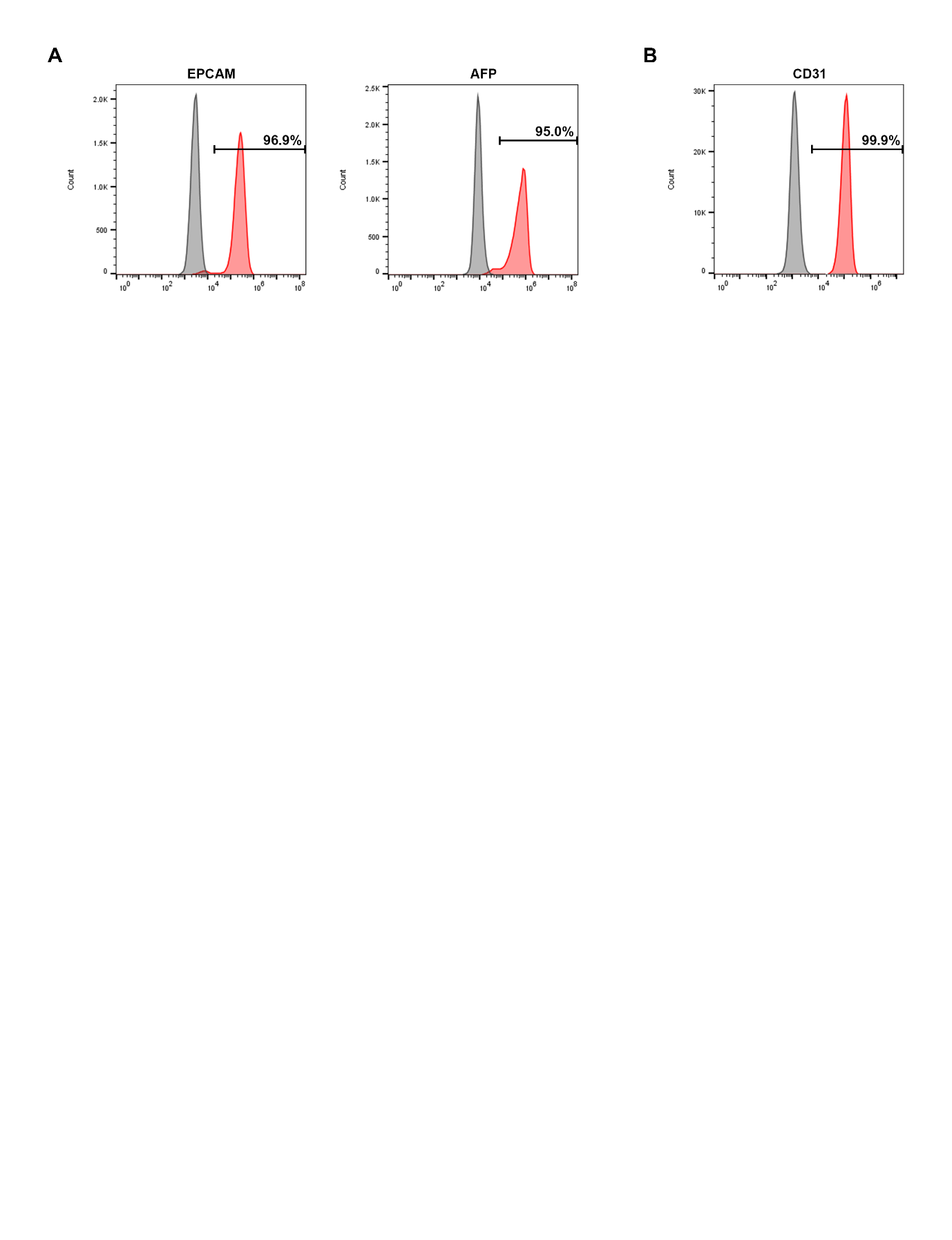


**Figure S1.** Flow cytometry analyses of EPCAM, AFP, and CD31 in hPSC-derived HE (**A**) and ECs (**B**).


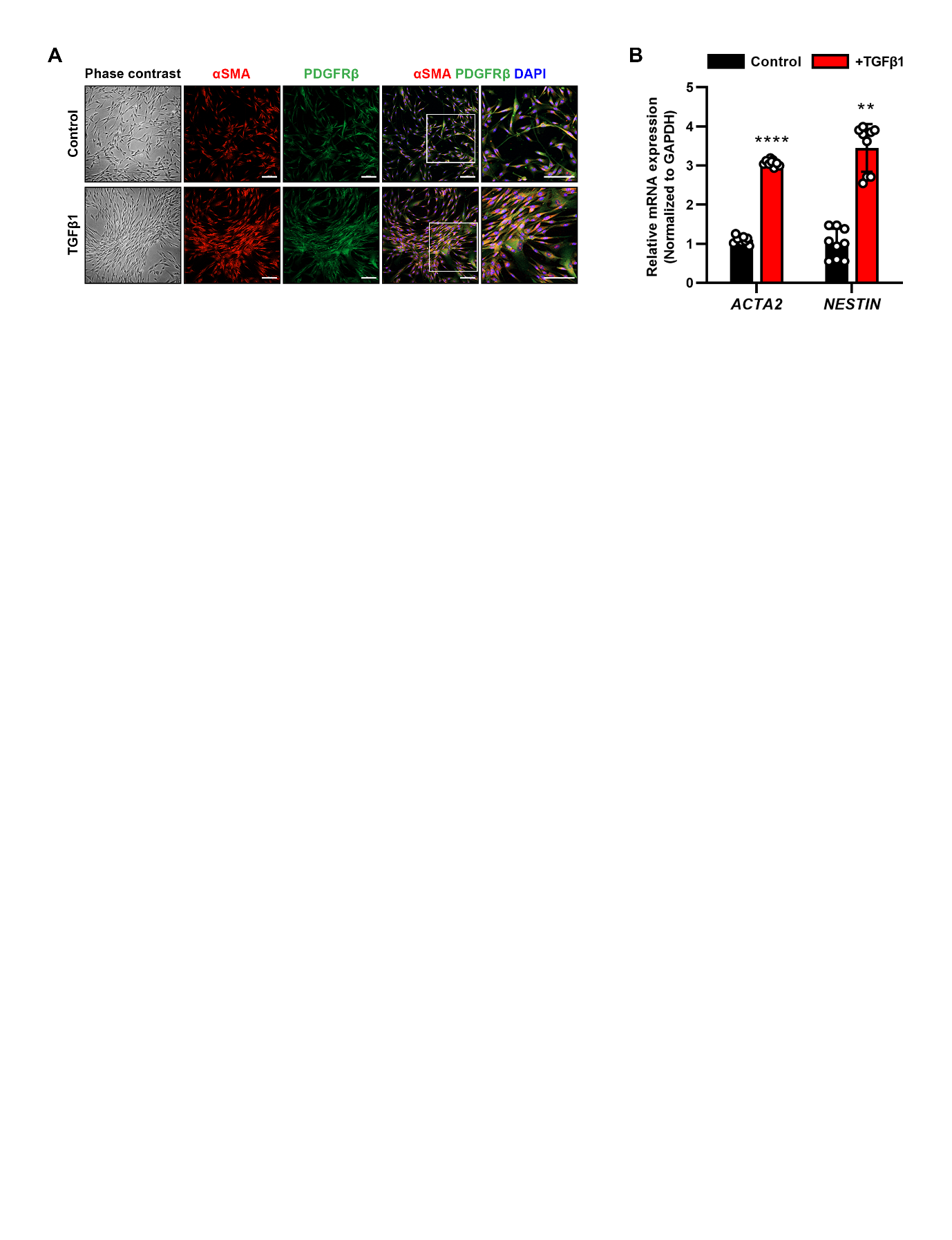
**Figure S2. Reactivity of hPSC-derived HscLCs to TGFβ1 treatment. A**, Phase-contrast and immunofluorescence images of a general marker of HSCs (PDGFRβ) and a key marker of activated HSCs (α-SMA) in hPSC-derived HscLCs after treatment with or without TGFβ1 for 48 h. Enlarged images of the boxed areas in α-SMA and PDGFRβ dual-staining images are shown separately in the right panels. Scale bar, 100 μm. **B**, qRT-PCR analysis of key markers of HSC activation *ACTA2* (which encodes α-SMA) and *NESTIN* in hPSC-derived HscLCs. Data are expressed as mean ± SD (n = 3 per group, normalized to *GAPDH*). ***P* < 0.01 and *****P* < 0.0001 by unpaired *t*-tests.

**
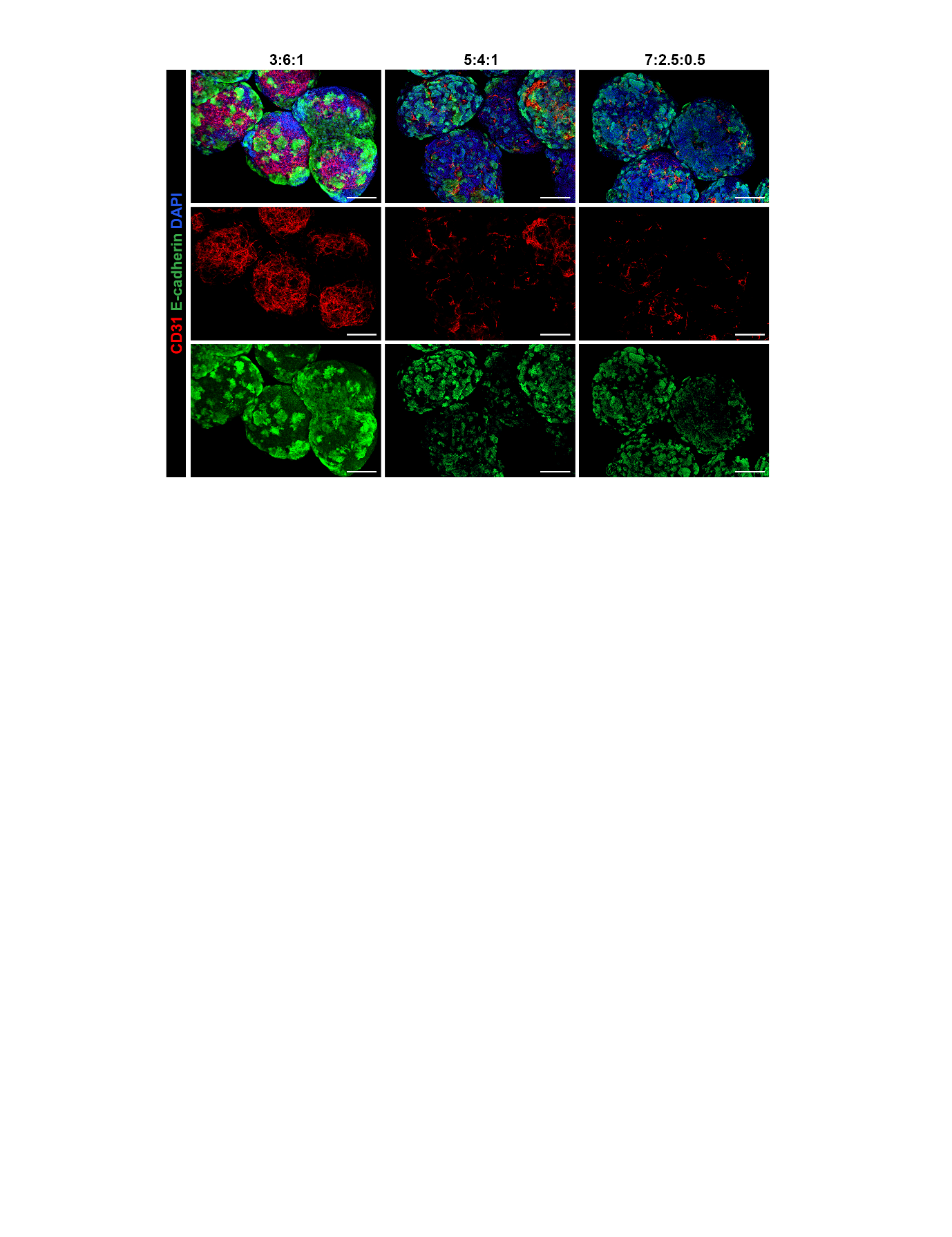
Figure S3.** **Comparison of mLOs assembled in different cell ratios for vascularization.** Three different cell types (HE:EC:HscLC) were assembled into mLOs in different ratios (3:6:1, 5:4:1, and 7:2.5:0.5) and differentiated for an additional 16 days. Note that CD31+ vascular networks were rarely detected when cell types were assembled in the ratios of 5:4:1 and 7:2.5:0.5. Scale bar, 200 μm.

**
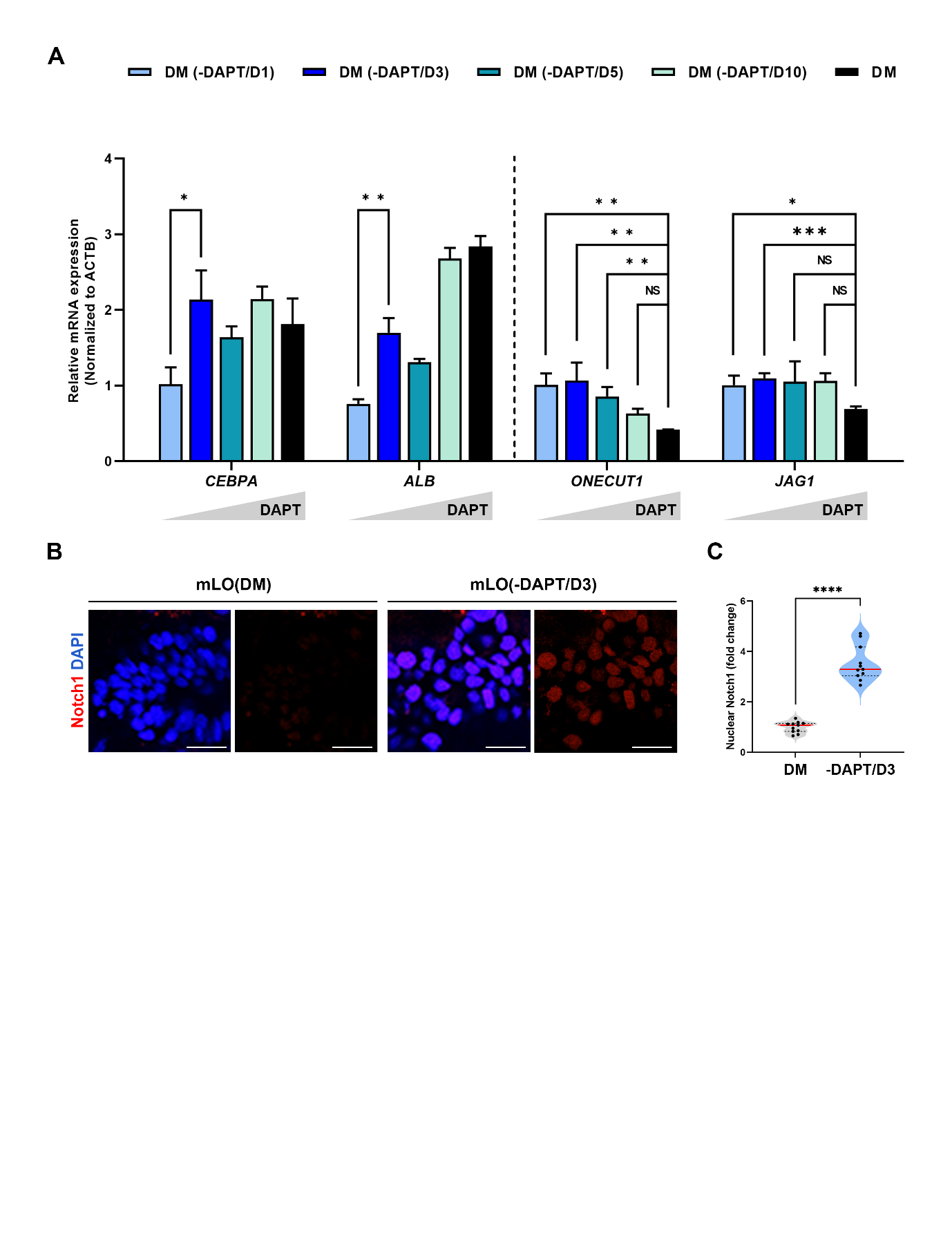
Figure S4.** **Comparison of hepatocyte- and bile duct-related gene expression pattern in mLOs after Notch modulation.** **A.** qRT-PCR analysis of mLOs showing the expression profile of genes associated with the formation of hepatocytes (*CEBPA* and *ALB*) and bile ducts (*ONECUT1* and *JAG1*) after treatment with DAPT at different times points of differentiation. Data are expressed as mean ± SD (n = 3 per group, normalized to *ACTB*). **p* < 0.05, ***p* < 0.01, and ****p* < 0.001 compared to DM-DAPT/D1 by unpaired *t*-tests. **B** and **C.** Immunofluorescence staining for Notch1 in the presence [mLO(DM)] or absence [mLO(-DAPT/D3)] of DAPT at day 8 of mLO generation (B). Scale bar, 20 μm. Quantification of cell nuclei labeled with Notch1 signals in mLOs (C). *****p* < 0.0001 compared to DM by unpaired *t*-tests.


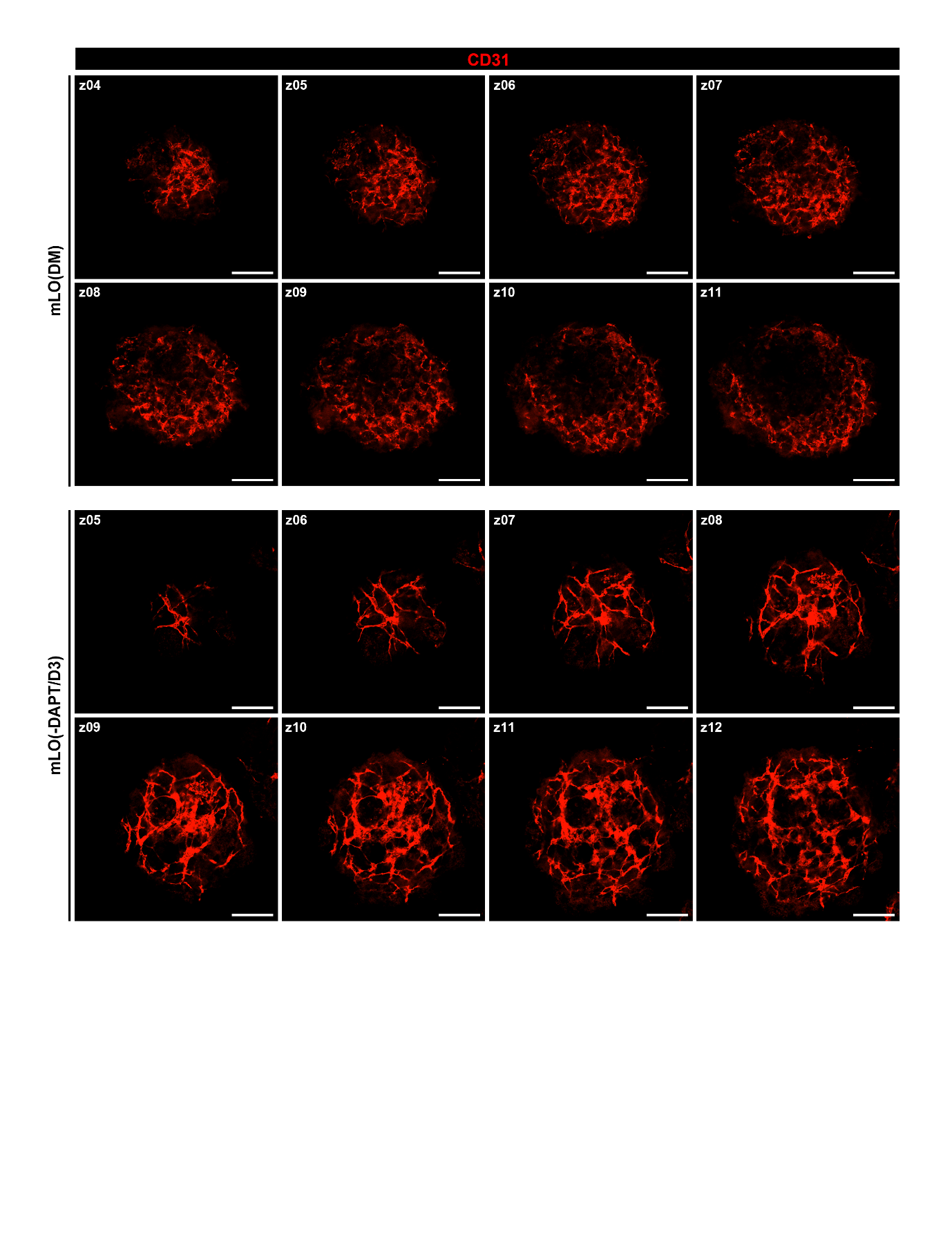


**Figure S5.** Serial z-scan confocal images of CD31+ vasculature in mLO(DM) and mLO(-DAPT/D3) after differentiation (day 16). Scale bar, 100 μm.

**
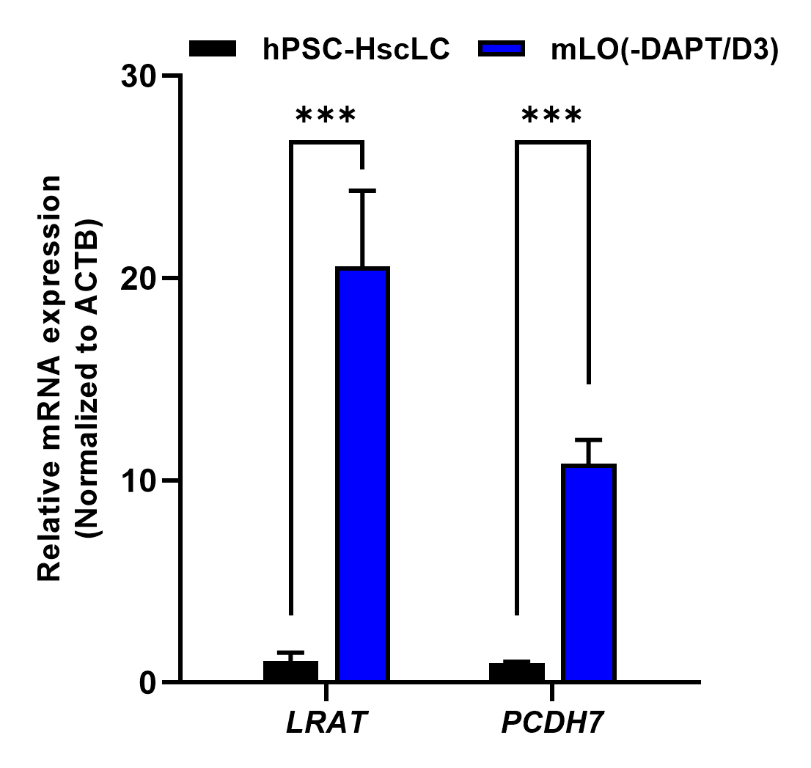
**

**Figure S6. Expression of HSC marker genes in hPSC-derived HscLCs and mLO(-DAPT/D3).** qRT-PCR analysis was performed to compare the expression levels of HSC-specific markers, LRAT and PCDH7, in hPSC-derived 2D HscLCs and mLO(-DAPT/D3). Data are expressed as mean ± SD (n = 3 per group, normalized to *ACTB*). ****p* < 0.001 by unpaired *t*-tests.

**
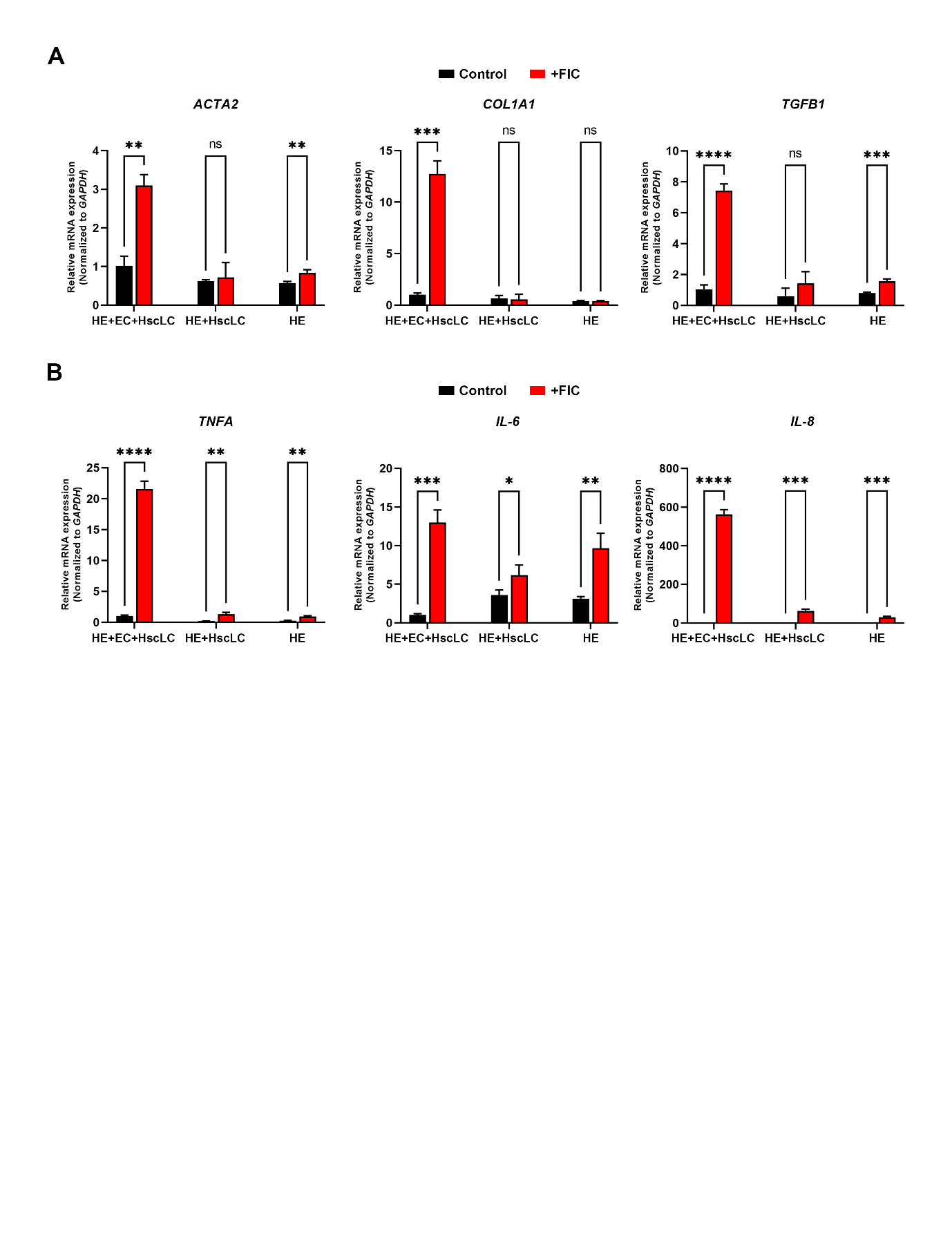
Figure S7. Responses of mLOs assembled in different combinations of cell types to FIC.** mLOs were generated by assembling three different combinations of cell types ((HE+EC+HscLC, HE+HscLC, and HE only) and exposed to FIC. qRT-PCR analysis was performed to compare the expression levels of genes related with the early fibrotic events (*ACTA2*, *COL1A1*, and *TGFB1*) and inflammatory cytokines (*TNFA*, *IL-6*, and *IL-8*). Data are expressed as mean ± SD (n = 3 per group). **p* < 0.05, ***p* < 0.01, ****p* < 0.001, and *****p* < 0.0001 by unpaired *t*-tests.

**Supplementary Video 1**. **3D rotation of stereo-projection of mLOs expressing the EC and hepatocyte makers.** A 3D rotation video of high-resolution confocal image of mLO(-DAPT/D3) expressing EC marker (CD31) and hepatocyte marker (HNF1β) at the end of differentiation (day 16).

**Supplementary Video 2**. **Live perfusion of luminal vasculature in mLO(-DAPT/D3)**. Time-lapse video created using a confocal image sequence of mLOs after *in vitro* live perfusion of rhodamine-conjugated UEA-I (red). Scale bar, 100 μm.

**References for supporting information**

1. Kim JH, Jang YJ, An SY, et al. Enhanced Metabolizing Activity of Human ES Cell-Derived Hepatocytes Using a 3D Culture System With Repeated Exposures to Xenobiotics. *Toxicol Sci*. Jan 2016;149(1):269. doi:10.1093/toxsci/kfv248

2. Park JY, Han J, Jung HS, et al. Synthetic probes for in vitro purification and in vivo tracking of hepatocytes derived from human pluripotent stem cells. *Biomaterials*. Nov 2019;222:119431. doi:10.1016/j.biomaterials.2019.119431

3. Lee G, Kim H, Park JY, et al. Generation of uniform liver spheroids from human pluripotent stem cells for imaging-based drug toxicity analysis. *Biomaterials*. Feb 2021;269:120529. doi:10.1016/j.biomaterials.2020.120529

4. Park CY, Kim DH, Son JS, et al. Functional Correction of Large Factor VIII Gene Chromosomal Inversions in Hemophilia A Patient-Derived iPSCs Using CRISPR-Cas9. *Cell Stem Cell*. Aug 6 2015;17(2):213-20. doi:10.1016/j.stem.2015.07.001

5. Son JS, Park CY, Lee G, et al. Therapeutic correction of hemophilia A using 2D endothelial cells and multicellular 3D organoids derived from CRISPR/Cas9-engineered patient iPSCs. *Biomaterials*. Apr 2022;283:121429. doi:10.1016/j.biomaterials.2022.121429

6. Coll M, Perea L, Boon R, et al. Generation of Hepatic Stellate Cells from Human Pluripotent Stem Cells Enables In Vitro Modeling of Liver Fibrosis. *Cell Stem Cell*. Jul 5 2018;23(1):101-113 e7. doi:10.1016/j.stem.2018.05.027

7. Orlova VV, van den Hil FE, Petrus-Reurer S, Drabsch Y, Ten Dijke P, Mummery CL. Generation, expansion and functional analysis of endothelial cells and pericytes derived from human pluripotent stem cells. *Nat Protoc*. 2014;9(6):1514-31. doi:10.1038/nprot.2014.102

8. Kumar A, D'Souza SS, Moskvin OV, et al. Specification and Diversification of Pericytes and Smooth Muscle Cells from Mesenchymoangioblasts. *Cell Rep*. May 30 2017;19(9):1902-1916. doi:10.1016/j.celrep.2017.05.019
